# A deep learning model to dynamically predict cancer-associated thromboembolism in large-scale healthcare systems

**DOI:** 10.1038/s41746-026-02730-2

**Published:** 2026-05-18

**Authors:** Tianshe He, Chunlei Zheng, Jennifer La, Dang Pham, Omid Jafari, Jamie Strampe, Karlynn Dulberger, Jaime Ramos-Cejudo, J. Michael Gaziano, Nhan V. Do, Vipul Chitalia, Katya Ravid, Ang Li, Nathanael R. Fillmore

**Affiliations:** 1 Boston Cooperative Studies Program, MAVERIC, VA Boston Healthcare System, Boston, MA USA; 2 Department of Psychiatry, New York University Grossman School of Medicine, New York, NY USA; 3 Department of Medicine, Boston University Chobanian & Avedisian School of Medicine, Boston, MA USA; 4 Brigham and Women’s Hospital, Harvard Medical School, Boston, MA USA; 5 Dan L Duncan Comprehensive Cancer Center, Baylor College of Medicine, Houston, TX USA; 6 Whitaker Cardiovascular Institute, Boston University Chobanian & Avedisian School of Medicine, Boston, MA USA

**Keywords:** Computational biology and bioinformatics, Diseases, Health care, Medical research, Risk factors

## Abstract

Venous thromboembolism (VTE) is a leading cause of preventable death among patients undergoing systemic treatment for cancer. Studies suggest that treatment strategies such as direct oral anticoagulant administration can significantly reduce the likelihood of VTE. Therefore, identifying people at high risk is of critical importance. Leveraging electronic health records (EHRs) from the U.S. Veterans Affairs (VA) healthcare system, we developed a transformer model to predict VTE risk in 80,808 cancer patients following the initiation of systemic treatment. The model uses longitudinal diagnostic codes, laboratory values, and demographic data. The proposed transformer model dynamically predicts VTE risk in 3-month quarterly intervals over the year following systemic treatment, achieving progressively improved performance across quarters (AUC: 0.68–0.77). The model is similarly performant on the external validation cohort from the Harris Health System (HHS) with 9752 patients (AUC: 0.68–0.74). By improving its predictions as a patient’s history evolves, this dynamic model surpasses prior static risk scores and better supports actionable decisions deeper into the treatment course.

## Introduction

Venous thromboembolism (VTE) is a major cause of death in patients receiving systemic therapy for cancer; however, it is a potentially preventable condition^1–3^. Clinical trials have established that interventions such as low-molecular-weight heparin (LMWH) or direct oral anticoagulants (DOAC) can substantially lower the incidence of VTE in high-risk patients^4,5^. These treatments are not only cost-effective but also demonstrate significant benefits regarding absolute risk reduction and their efficacy-to-safety ratio^6–10^. Consequently, the ability to identify patients at risk of VTE in a dynamic, real-time manner can enable life-saving and cost-effective prophylactic measures. The success of such strategies hinges on the accurate stratification of patients by VTE risk^11^ and the timely implementation of treatment^12^.

Several risk assessment models (RAMs) have been created to detect VTE risk^13–18^. The Khorana Score, a widely used tool, assesses risk based on cancer type and laboratory values but has notable limitations in sensitivity and discriminatory power. Furthermore, its application is restricted to patients with solid tumors and lymphomas who are receiving chemotherapy^19^. More recently, Li et al. developed EHR-CAT, an updated RAM that integrates 11 predictors that are readily available in clinical practice^20^ and demonstrates increased discrimination and coverage in multiple settings^21,22^.

Despite these advances, predicting VTE in cancer patients remains a challenge, particularly for cases occurring more than 6 months after the initiation of systematic treatment^20,23^. A patient’s medical history is critical for assessing future disease risks, yet existing models, while attempting to use this information through aggregation, often fail to capitalize on the rich longitudinal data within EHRs. Model development is further complicated by irregularities in time and data missingness.

In recent years, neural networks like transformers and other attention-based models^24^ have emerged as a powerful solution to these challenges, capable of learning patterns from minimally processed, noisy, and irregular time-series data^25^. Sequential frameworks, including recurrent neural networks^26^, long short-term memory^27^, and transformers^28,29^, have been successfully applied to cancer prediction using EHRs. While some of these machine learning methods have been used for dynamic VTE prediction^23,30–36^, they are often constrained to specific disease scenarios, require specialized biomarkers like d-dimer, or focus narrowly on short-term outcomes. A predictive model for cancer-associated VTE risk that fully leverages the time-varying characteristics from routinely generated EHR trajectories has yet to be developed.

In this study, we developed and validated a transformer model that uses trajectories of disease and laboratory data to predict VTE incidence within one year of systemic treatment initiation for cancer patients in the nationwide Veterans Affairs (VA) healthcare system^29^, and we further externally validated this model in data from the Harris Health System (HHS). The proposed artificial intelligence model is designed to achieve improved predictive performance by integrating additional information as it becomes available deeper into a patient’s treatment course, thereby allowing interventions to be more precise and delayed until they are most necessary.

## Results

### Patient characteristics

The primary derivation cohort consisted of 80,808 cancer patients from the nationwide VA healthcare system who initiated systemic therapy between 2011 and 2020. The cohort was predominantly male (95.1%), white (71%), with a median age at cancer diagnosis of 67. Lung and prostate cancer were the most frequent cancer types, and chemotherapy was the most common treatment. Within one year of starting systemic treatment (the treatment index date), 4873 patients (6.0%) developed VTE. The incidence of VTE decreased over time, with 3.2% of cases occurring in the first quarter after treatment index date (Q1: 0–3 months), 1.7% in Q2 (3–6 months), 1.1% in Q3 (6–9 months), and 0.9% in Q4 (9–12 months) (Table 1). An additional internal temporal validation cohort of 3303 patients from the VA with treatment index dates between 2021 and 2022 was established for subsequent model evaluation, with similar characteristics to those of the main cohort (Table S.I.2).

**Table 1:** Patient characteristics of the primary cohort (treatment index dates 2011–2020)

	Did not experience VTE within 1 year of treatment	Experienced VTE within 1 year of treatment
**Sample Size** (0–1 year past treatment), n (%)	75,935 (94.0%)	4873 (6.0% prevalence)
*Q1* (0–3 months past treatment)	78,241 (96.8%)	2567 (3.2% incidence)
*Q2* (3–6 months)	69,812 (98.3%)	1172 (1.7% incidence)
*Q3* (6–9 months)	63,264 (98.9%)	649 (1.1% incidence)
*Q4* (9–12 months)	56,599 (99.1%)	485 (0.9% incidence)
**Demographics**		
**Age at treatment index date**, median (IQR)	67.4 [62.4, 72.8]	66.8 [61.7, 71.9]
**Sex**, *n* (%) Female	3708 (4.9%)	241 (4.9%)
**Race**, *n* (%)		
White	57,614 (75.9%)	3669 (75.3%)
Black or African American	15,524 (20.4%)	1051 (21.6%)
American Indian or Alaskan Native	469 (0.6%)	27 (0.6%)
Asian, N. Hawaiian, Pacific Islander	544 (0.7%)	32 (0.6%)
Unknown	1784 (2.3%)	94 (1.9%)
**Ethnicity** (Hispanic/Latino), *n* (%)	2432 (3.2%)	149 (3.1%)
**BMI Category**, n (%)		
Underweight or healthy weight (0–24.9)	22,116 (29.2%)	1290 (26.5%)
Overweight (25–29.9)	25,984 (34.2%)	1680 (34.5%)
Obese (30–34.9)	17,037 (22.4%)	1090 (22.4%)
Obese II (≥35)	10,745 (14.2%)	813 (16.4%)
**Cancer type (most common)**, *n* (%)		
Lung	15,799 (20.8%)	1407 (28.9%)
Prostate	13,062 (17.2%)	245 (5.0%)
Liver	4040 (5.3%)	107 (2.2%)
Bladder	3510 (4.6%)	237 (4.9%)
Multiple Myeloma	2596 (3.4%)	192 (3.9%)
Esophagus	2296 (3.0%)	277 (5.7%)
Pancreas	2112 (2.8%)	308 (6.3%)
**Cancer Stage**, *n* (%)		
Early (I or lower)	15,474 (20.4%)	760 (15.6%)
Intermediate (II or III)	28,408 (37.4%)	1692 (34.7%)
Advanced (IV)	32,053 (42.2%)	2421 (49.7%)
**Treatment Type** (not mutually exclusive), *n* (%)		
Chemotherapy	52,771 (69.5%)	4149 (85.1%)
Endocrine therapy	13,280 (17.5%)	244 (5.0%)
Immunotherapy	1916 (2.5%)	168 (3.4%)
Targeted therapy	15,281 (20.1%)	956 (19.6%)
**Follow-up times (days)**, mean [IQR]		
Days to VTE from treatment		113 [40,168]
Days to treatment from cancer diagnosis	73 [27,92]	57 [25,72]

An external validation set was derived, comprised of cancer patients from the Harris Health System (HHS), an integrated safety-net health care system with two medical centers and 18 outpatient clinics for patients from diverse demographic backgrounds in the Houston metropolitan area. The HHS cancer cohort was derived in the same way as that of the primary VA cohort with treatment index dates from 2011–2020, with 9752 total patients and 721 VTE cases within one year of treatment initiation^20^. Patients from HHS were younger (median age, 55 years), mostly (57%) female, and the majority non-white (52% Hispanic, 26% Black, 15% White), contrasting with those from the VA healthcare system. Lung and prostate cancers, the most common cancer types in the VA healthcare system, were well-represented in the HHS. In contrast, breast cancer was the most common in HHS but minimally represented in the VA owing to demographic differences. HHS patients generally experienced shorter times from treatment to diagnosis and shorter times to VTE from treatment initiation (84 days) compared to those in the VA (113 days) (Table S.I.3). The median number of EHR events for each patient before treatment initiation was 103 in HHS compared to 157 in the VA.

### Patient EHR trajectories

**Fig. 1:**
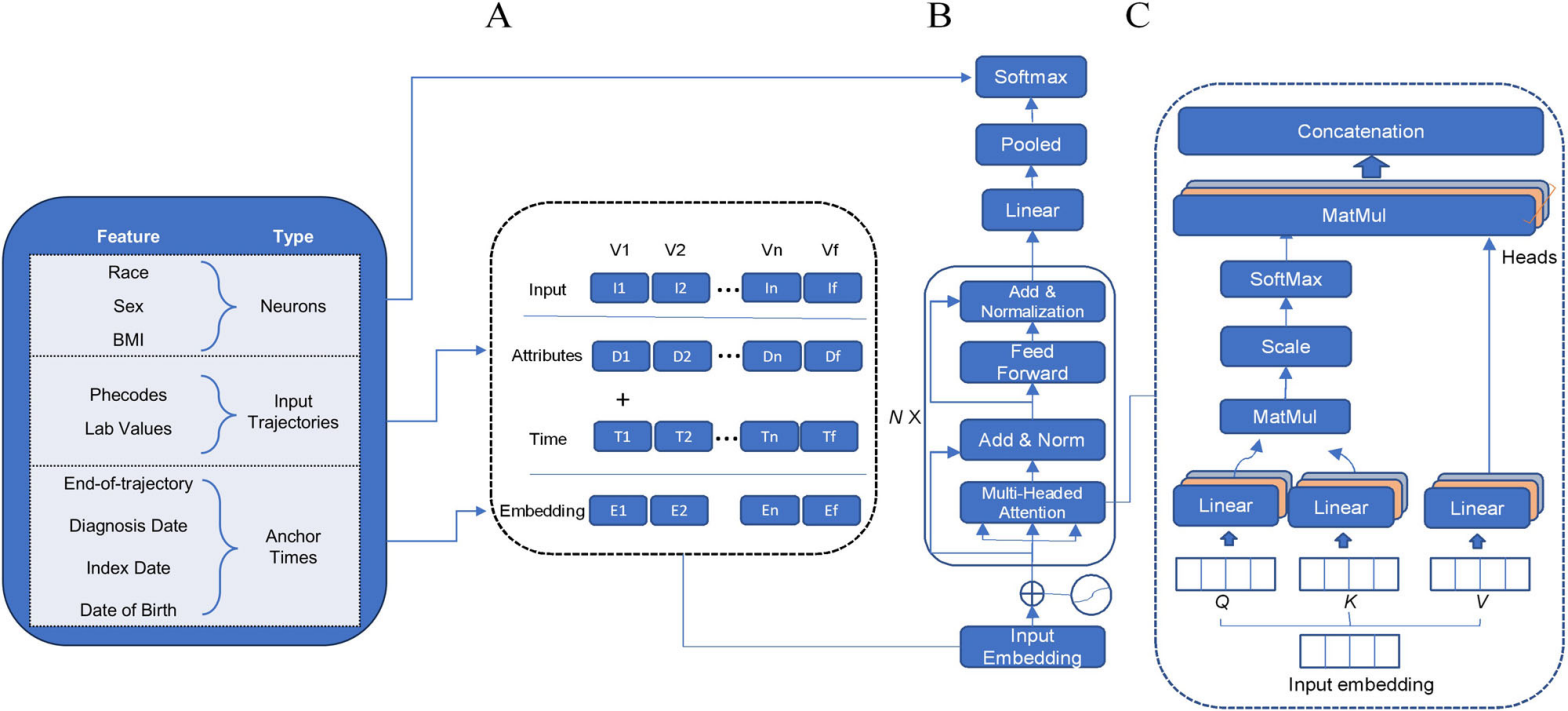
Transformer architecture. **A** Diagram for the input embedding. Phecodes, lab values, and associated timestamps were included in the embedding layer. The composition of these embeddings forms the latent contextual representation of the patient’s diagnostic history. **B** Overall transformer architecture. Embedded data are fed into multi-headed transformer layers, where long-time dependencies of diseases and labs are learned. Then, one or more linear layers are added to learn more features. The Softmax layer is used to predict VTE risk. **C** Details for the multi-headed attention layer in the transformer. Multi-heads were used in this transformer since it can learn different disease dependencies. Query, key, and value are created by input embedding, and dot product attention will be used to generate attention scores, which will be added to the value to generate the attention layer. Multi-attention layers will be finally concatenated to produce the final attention layer.

**Fig. 2:**
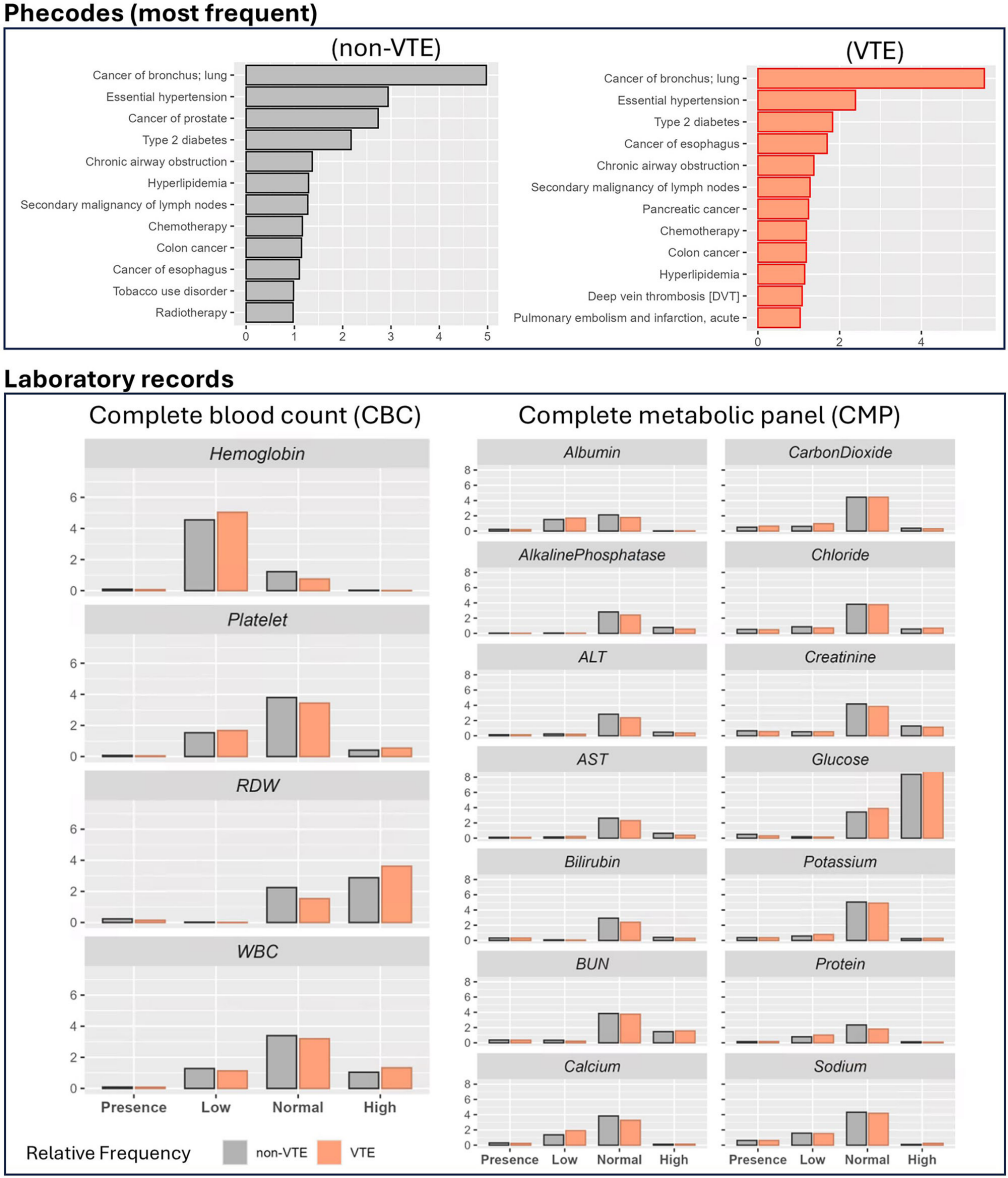
Characteristics of phecode and lab input features. (Top) Relative frequencies (% of total phecodes stratified by outcome) of the most frequent phecodes for patients who develop VTE within 1 year of treatment index date (right) and those who do not (left) in the primary cohort. DVT and PE phecodes among VTE patients indicate prior VTEs. (Bottom) Relative frequencies of CBC (left) and CMP (right) laboratory records for the total cohort. Lab values are grouped by levels relative to the normal range: *low, normal, high, and (unknown) presence*. Frequencies (%) are calculated by total lab inputs stratified by VTE outcome.

Longitudinal patient trajectories comprised of time-stamped diagnostic codes (phecodes) and laboratory records from the patient’s medical history are constructed to predict VTE risk using a transformer encoder architecture **(**Fig. 1). The primary input features included 1,862 distinct phecodes, which are synoptic concepts derived from diagnostic ICD (International Classification of Diseases) codes^37,38^, and 72 distinct laboratory combinations (*unknown, low, normal, high* for each of the 18 labs) derived from complete blood cell count (CBC) and complete metabolic panel (CMP) tests (Fig. 2). Sex^39^, race, ethnicity, and body mass index (BMI) at treatment index date were included as static covariates. Cancer diagnosis dates and treatment index dates were used as anchor times to account for the relative positional encoding of tokens with respect to these points in the patient’s history.

**Fig. 3:**
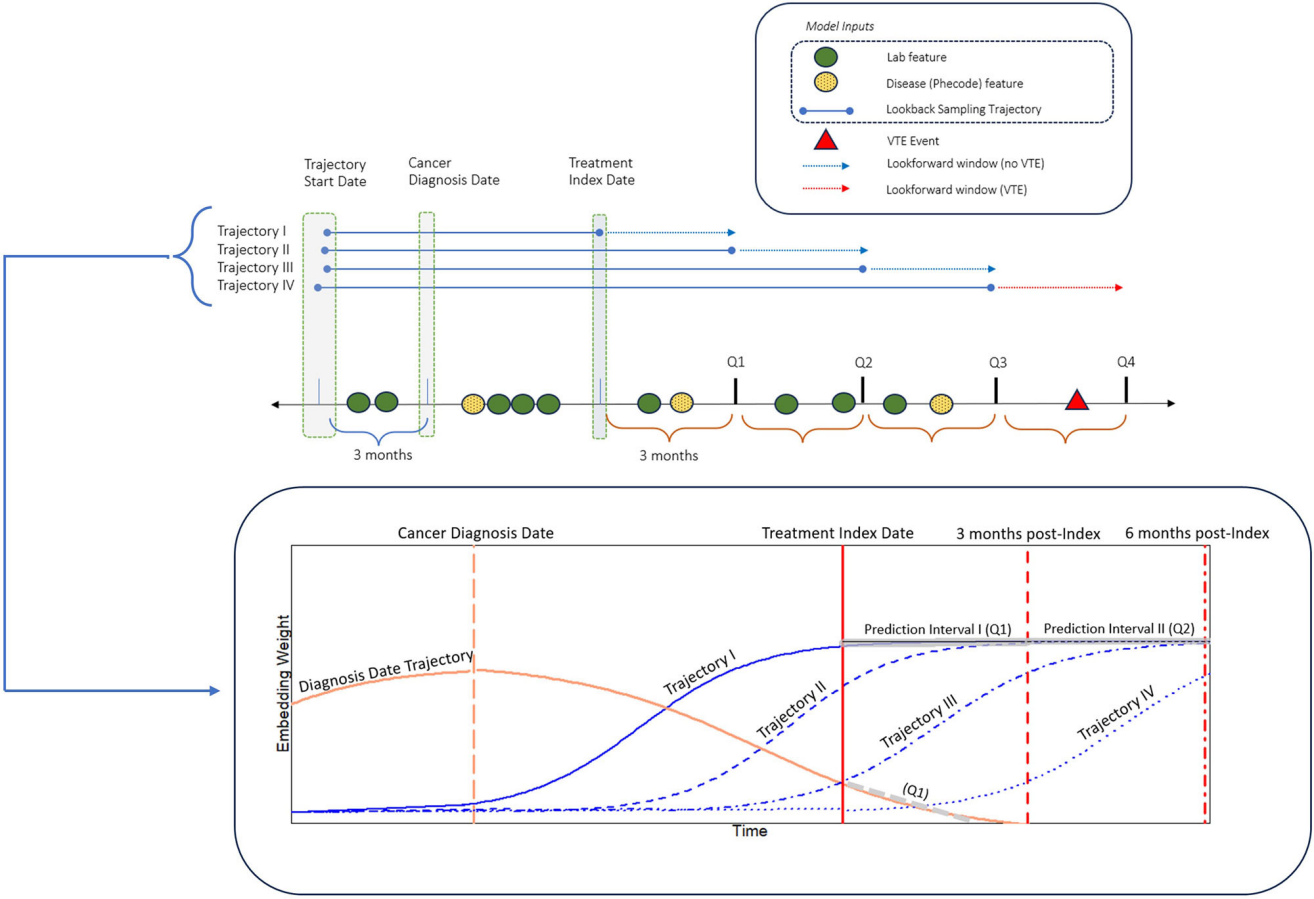
(Top) Schematic diagram of proposed model design. (Bottom) relative contribution of time-embeddings (time-to-end) and time-from-diagnosis embeddings; embeddings about the treatment index date are also used in the model, but are not shown. Age embeddings are also not shown.

**Fig. 4:**
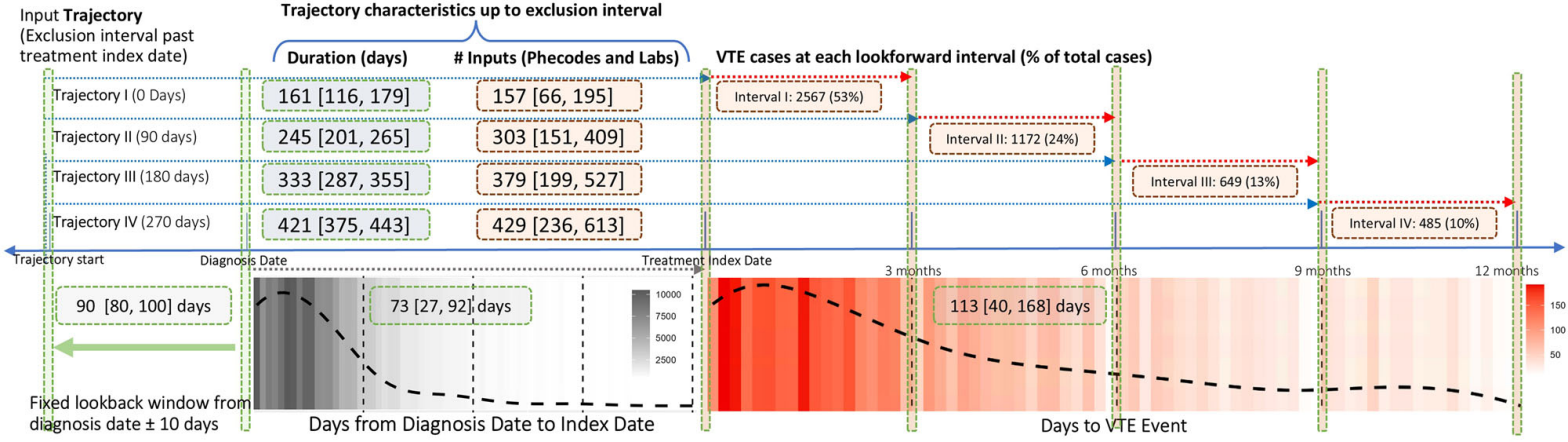
Input trajectory and prediction interval characteristics for each of the four intervals (Top left). Median length of input trajectories in duration and number of input events. (Top right) Number of VTE cases for prediction intervals Q1 (0–3 months), Q2 (3–6 months), Q3 (6–9 months), Q4 (9–12 months). (Bottom left) Median duration of interval before diagnosis date (fixed at 90 ± 10 days) and duration distribution between diagnosis date to treatment index date (median 73 days with IQR [27,92] for all input trajectories. (Bottom right) Distribution of duration from treatment index date to VTE event among those who experience VTE (median 113 days with IQR [40,168]).

A schematic diagram of the study’s dynamic cohort prediction design^40^ is illustrated in Fig. 3. The model uses four distinct input trajectories (I-IV) of phecodes and labs to predict VTE risk in four consecutive 3-month look-forward windows (Q1-Q4) following the initiation of systemic treatment. Based on previous estimates of cancer onset timing^41–43^, all input trajectories began approximately 90 days prior to the cancer diagnosis date. Trajectories I-IV ended at 0, 3, 6, and 9 months after the treatment index date, respectively. In the primary derivation cohort (VA), each patient had average durations of 5, 8, 11, and 16 months and contained an average of 157, 303, 379, and 429 input events (Fig. 4). If a patient experienced VTE at a given quarter, they were considered a positive case for that quarter and excluded from subsequent prediction intervals.

### Dynamic risk prediction using a transformer

The transformer model processes patient EHR trajectories to predict dynamic VTE risk through three key mechanisms: (1) encoding temporal sequence information, (2) embedding input trajectory events, and (3) multi-head attention. Our embedding approach integrates patient-specific anchor times with evolving patient trajectories to process temporally dynamic representations of clinical history. Multi-head attention makes use of these representations by learning pairwise relationships among all input events and capturing complex dependencies across patient-trajectory combinations. Model architecture is described in detail in **Methods**.

### Model performance

The model’s performance was evaluated on a held-out test set that consisted of 20% of the full cohort (details in **Methods** and Table S.I.2) and the separate temporal validation cohort (Table S.I.1). On the primary test set, the transformer model demonstrated progressively improved performance as more longitudinal data became available. It achieved AUCs of 0.68 (95% CI: 0.66–0.70) for Q1, 0.68 (0.65–0.71) for Q2, 0.71 (0.67–0.75) for Q3, and 0.77 (0.72–0.81) for Q4. The model’s performance was consistent on the internal temporal validation set (Q1-Q4 AUC: 0.65, 0.60, 0.76, 0.76) and the HHS external validation cohort (Q1-Q4 AUC: 0.68, 0.70, 0.71, 0.74) (Table 2).

**Table 2:** Validation Performance

	Test (Treatment index 2011–2020)			Temporal Validation (Treatment index 2021–2022)			External Validation (HHS) (Treatment index 2011–2020)		
*Quarter*	*n*	*Cases (%)*	*AUC*	*n*	*Cases (%)*	*AUC*	*n*	*Cases (%)*	*AUC*
*Q1*	16132	480 (3.0%)	0.679	3303	132 (4.0%)	0.653	9752	378 (3.9%)	0.683
*Q2*	14226	220 (1.5%)	0.682	2880	48 (1.7%)	0.601	8537	184 (2.2%)	0.703
*Q3*	12829	153 (1.2%)	0.707	2639	16 (0.6%)	0.761	7244	93 (1.3%)	0.709
*Q4*	11681	101 (0.9%)	0.765	2453	17 (0.7%)	0.760	5962	66 (1.1%)	0.738
*Overall*		954 (5.9%)	0.733		213 (6.4%)	0.758		721 (7.4%)	0.739

Sample size, case rate (incidence at each quarter and overall 1-year prevalence), and AUCs of the transformer in (1) the (primary) test set with treatment index dates between 2011–2020, (2) internal temporal validation set with index dates from 2021–2022, and (3) external validation set from HHS, with treatment index dates also from 2011–2020.

**Fig. 5:**
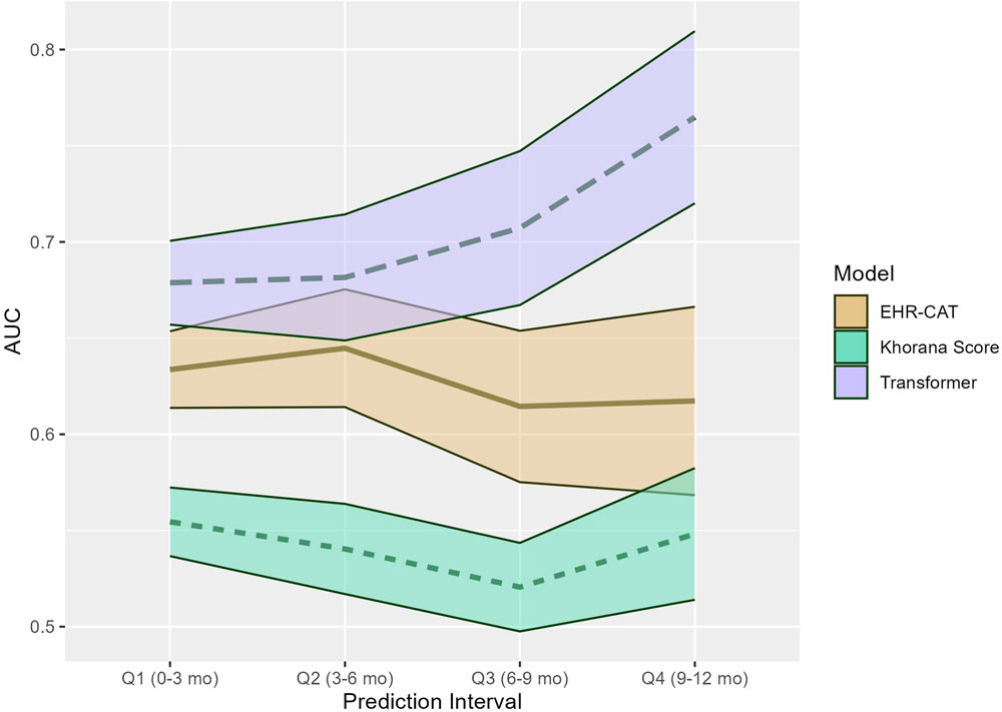
Model Comparison Results from the proposed model compared with the EHR-CAT risk assessment and Khorana Score. The AUCs for the transformer model are 0.679, 0.682, 0.707, and 0.765, respectively, across the four prediction intervals. AUCs for EHR-CAT (RAM) score are 0.634, 0.645, 0.613, 0.617. AUCs for the Khorana Score are 0.555, 0.540, 0.520, and 0.548. At each of the consecutive prediction windows, 95% confidence intervals for predictions from the transformer model are [0.66, 0.70], [0.65, 0.71], [0.67, 0.75], and [0.72, 0.81]. Confidence intervals for AUCs are calculated from bootstrapping using the pROC package in R^77^. 95% CIs for EHR-CAT score are [0.61, 0.65], [0.61, 0.68], [0.57, 0.65], [0.57, 0.67], and for Khorana score are [0.54, 0.57], [0.52, 0.56], [0.50, 0.54], [0.51, 0.58].

The transformer model significantly outperformed two established risk models, EHR-CAT and the Khorana Score (Fig. 5). Its advantage was most pronounced in later quarters; for instance, the transformer’s Q4 AUC of 0.77 was substantially higher than that of EHR-CAT (0.62) and the Khorana Score (0.55).

Furthermore, the model showed improved recall (sensitivity) for identifying at-risk patients. Compared to EHR-CAT, the transformer model’s recall increased by 11% and 9% in the first two quarters and by 34% and 50% in Q3 and Q4, respectively, with even greater improvements over KS (Table 3). Associated efficiency metrics (number needed to evaluate: NNE^44^, number needed to screen to prevent one VTE: NNS-V^45^) revealed similar patterns (Table S.IX.1). Labor-time per VTE detected under an example precision-augmented screening workflow (Fig. S.IX.1) is quantified in Table S.IX.2, ranging from 4.0 labor-hours in Q1 to 9.8 labor-hours in Q4. Finally, the model was well-calibrated, with predicted probabilities closely matching the observed VTE frequencies (Fig. S.II.1–3**)**.

**Table 3:** Confusion Matrices for transformer post-calibration and EHR-CAT and Khorana scores

**Transformer Model**											
*Q1* (0-3)	VTE-	VTE+	*Q2* (3-6)	VTE-	VTE+	*Q3* (6-9)	VTE-	VTE+	*Q4* (9-12)	VTE-	VTE+
Pred-	6697	86	Pred-	6965	52	Pred-	6443	34	Pred-	6603	16
Pred+	8955	394	Pred+	7041	168	Pred+	6233	119	Pred+	4977	85
Re/Pr	0.82	0.042	Re/Pr	0.76	0.023	Re/Pr	0.78	0.019	Re/Pr	0.84	0.017
**EHR-CAT Score** (at matching prediction intervals)											
*Q1* (0-3)	VTE-	VTE+	*Q2* (3-6)	VTE-	VTE+	*Q3* (6-9)	VTE-	VTE+	*Q4* (9-12)	VTE-	VTE+
Pred-	8195	123	Pred-	8257	66	Pred-	8124	63	Pred-	7717	44
Pred+	7457	357	Pred+	5749	154	Pred+	4552	90	Pred+	3863	57
Re/Pr	0.74	0.045	Re/Pr	0.70	0.026	Re/Pr	0.59	.019	Re/Pr	0.56	0.014

**Khorana Score** (at matching prediction intervals)											
*Q1* (0-3)	VTE-	VTE+	*Q2* (3–6)	VTE-	VTE+	*Q3* (6–9)	VTE-	VTE+	*Q4* (9–12)	VTE-	VTE+
Pred-	14,326	387	Pred-	13,101	188	Pred-	11,999	139	Pred-	10,793	87
Pred+	1326	93	Pred+	905	32	Pred+	676	14	Pred+	788	14
Re/Pr	0.19	0.066	Re/Pr	0.15	0.034	Re/Pr	0.09	0.020	Re/Pr	0.14	0.017

For the transformer model, thresholds for a positive prediction (VTE+) are chosen from the incidence rates of the calibration (development) set at 3.4%, 1.9%, 1.1%, and 0.9%. An EHR-CAT or Khorana score of 3 or over corresponds with a positive prediction. Recall (Re) and Precision (Pr) are also provided for each model.

### Model performance accounting for the competing risk of death

Competing risks analyses were conducted to account for the high mortality rates among cancer patients, not attributable to VTE. In the primary test set, deaths totaled 1385 (9%), 2079 (15%), 2369 (18%), and 2328 (20%), respectively, in prediction windows Q1-Q4. We used cause-specific AUCs^46^ (CS-AUCs) to evaluate the model discrimination for VTE, accounting for the competing risk of death using inverse probability of censoring weighting (IPCW). CS-AUCs ranged from 0.70 (95% CI: 0.67–0.73) at the midpoint (45 days) of Q1 up to 0.81 (0.76–0.86) at the midpoint of Q4, attenuating to 0.76 (0.72–0.81) at 90 days (Fig. S.VII.1). Calibration plots that accounted for competing risks were constructed using cumulative incidence functions (CIFs) for VTE. These plots showed that the model predictions remained well-calibrated even in the presence of a high mortality rate (Fig. S.VII.2**)**.

### Hyperparameter configuration and sensitivity analyses

The optimal hyperparameter configuration used 8 attention heads, 1 hidden layer with a dimension of 128, and a maximum patient trajectory length of 800 tokens. The trajectory start date of 90 days prior to cancer diagnosis was selected based on optimal performance, but also dovetail with the initial hypothesis based on cancer onset timing^41–43^. Pad sizes of 400 were selected to best balance input retention and training efficiency. Padding standardizes the length of patient history records by appending null (empty) entries to shorter records, allowing the model to standardize the length of patient history records for uniform input sequences during training. The full set of hyperparameters is provided in Table S.II.1. Sensitivity analyses explored variations in trajectory length and model complexity parameters and showed that increasing these did not improve prediction performance, thereby supporting the parsimony of the selected architecture. Ablation analysis confirmed the value of each input data type. When phecodes, labs, static covariates, or anchor time embeddings were individually removed from the model, the evaluation AUCs decreased across all four quarters, underscoring the importance of the different types of features. Ablation analysis confirmed that all input data types, as well as anchor times, were indispensable in model performance (Tables S.II.3–6). Additionally, models fixed at Q1 weights applied to Q2-Q4 for both VA test and HHS validation sets revealed similar performance as that of Q1 as well as the EHR-CAT score (Table S.II.7).

### Subgroup analyses across differing healthcare systems

The transformer performed consistently across the most common cancer and treatment types (Table S.III.1**)**. Notably, performance was particularly strong among non-white and black patients, where AUCs in later quarters reached as high as 0.83 (95% CI: 0.76–0.90) (Table S.III.2). Subgroup analysis in HHS revealed similar patterns of increasing predictive performance as in the primary test set from Q1-Q4. Notably, the AUCs of the *female* and the *under 55* subgroups increased monotonically from 0.69–0.77 and 0.67–0.78 from Q1-Q4, despite disproportionate underrepresentation in the derivation cohort (Table S.III.3). Performance in the breast cancer subgroup in HHS also showed similar trends (Table S.III.4)

### Identifying clinically interpretable features

Model attribution analyses confirmed that diagnoses such as brain cancer, lung cancer, and secondary metastasis to the liver were assigned high importance, which was consistent with established clinical risk factors^47–49^. Additionally, known indicators for VTE, such as *low albumin*^50^ and *shortness of breath*^51^ were also captured respectively at Q1-Q3 and Q4 (Figs. S.VI.1-2**)**.

## Discussion

This study introduces a deep learning model that dynamically predicts the risk of cancer-associated VTE using longitudinal EHR data. The model significantly outperforms existing risk assessment models, particularly for predictions made more than six months after the initiation of systemic therapy. As its performance improves over the course of a patient’s treatment, the transformer achieved AUCs up to 0.77 in the primary test set and increased patient identification (recall) by up to 50% in later quarters compared to existing methods. The model showed consistent performance in internal temporal validation as well as external validation in a health system with substantial cohort differences.

The model’s improved performance can be attributed to its time-varying design and the transformer architecture’s ability to model complex, non-linear relationships over time. Timing is of critical importance in modeling cancer-associated VTE, since prognoses vary for a patient in different periods after initiating cancer treatment. Unlike static risk scores, which lose accuracy over time, the transformer’s use of multi-headed attention layers and positional encodings allows it to adaptively forecast VTE risk, capturing shifts in likelihood as a patient’s clinical history evolves. This dynamic updating capability is the model’s key advantage; for instance, if a patient remains VTE-free through the first six months of cancer treatment, the model adjusts the subsequent risk forecast accordingly, a patient-specific adjustment that simpler models cannot make. This dynamic approach parallels real-world clinical timelines, offering practitioners a tool to inform time-sensitive decisions, such as when to initiate anticoagulant therapy*.*

Our model was designed to maximize recall to prioritize the identification of true positive cases over minimizing false positives. This reflects the clinical priority of preventing a deadly condition where cost-effective prophylactic therapies are available^6–10^. Low precision is a known challenge for existing VTE risk tools due to the rarity of cases, including EHR-CAT and Khorana scores (KS), as reflected in the results of this study. However, the NNE derived from the transformer’s precision is concordant with screening procedures for common cancers^44^. Since KS is recommended in the American Society of Hematology (ASH) 2021 guidelines for management of VTE^52^, both EHR-CAT and the transformer model could plausibly be incorporated into clinical practice guidelines. NNS-V shows significant advantages in transformer compared to EHR-CAT and KS, and is consistent with those of cancer screenings for one preventable death^45^. This comparison is inexact as VTE’s direct mortality impact is difficult to isolate; however, cancer-related VTE markedly worsens outcomes, with one-year survival of ~12% after an event versus ~36% with cancer alone^53^.

A 3-month prediction window was selected to balance clinical urgency with feasibility. Since VTE events can arise quickly, this time window allows for timely identification and preventive action. The 3-month window also allows enough time for the deployment of prophylactic intervention. Short-term predictions also require shorter input trajectories. Sensitivity analyses showed that the model’s quarterly predictions do not require extensive historical data before the cancer diagnosis, as longer lookback windows did not improve performance. The model requires only a short three-month lookback window from initial cancer diagnosis and as few as five health records, suggesting that it can be adapted to settings with limited or fragmented data. This flexibility was a key factor that allowed the model to be validated on the HHS dataset.

Originally developed and internally validated within the VA healthcare system, the transformer was successfully applied to the HHS dataset. Despite substantial heterogeneity between the systems due to differences in patient demographics, coding practices, and data representation, the model was demonstrably transferable. The synoptic representations chosen for token vocabularies (phecodes instead of ICD, broad lab categories in relation to normal ranges) aid in this translation and allow for a *common corpus* across different healthcare systems.

External validation on the HHS dataset showed consistent performance improvements from Q1 to Q4. The HHS cohort, with greater racial diversity, balanced sex distribution, and a younger age profile compared to the VA cohort, offered a broader and more representative testing ground. Subgroup analyses further demonstrated the model’s robustness, with consistent performance across granular demographic strata. AUCs in HHS subgroups increased steadily over time and reflected overall trends in both cohorts. This pattern was especially salient among female and younger patients (under 55) as well as those with breast cancer in the HHS, despite their underrepresentation in the VA training data. Even in demographic groups with lower case rates in later quarters, narrow AUC confidence intervals yielded stable and reliable performance, reinforcing the model’s generalizability within the diverse HHS cohort.

The inclusion of laboratory values alongside diagnostic codes also systematically improved model performance at all prediction windows. Though there is potential overlap between these two categories (e.g., *anemia* and *low platelet count*), their combination yields added predictive value, as shown in ablation analysis. The transformer may detect early VTE signals from subtle changes in lab results or new diagnoses that were missed by physicians. The model design also treats missing laboratory values as informative features, preserving the implicit signal that a clinically stable patient may not have required testing. Neither the VA nor HHS datasets contain missing static variables; however, if external validation datasets contain missing values in these fields, imputation strategies may be employed. When a patient’s medical records are missing from a gap in recording, e.g., not being present at all for a given period, the transformer can implicitly impute hidden states from learned patterns — one of the main advantages of the deep learning approach.

The model’s reliance on temporal data introduces potential for technical bias. We mitigated this by using fixed 90-day prediction windows^40^ and anchoring embeddings to fix timepoints for each patient (diagnosis date and treatment index date). The model’s consistent performance in temporal validation, along with low correlations between predicted probabilities and time-to-VTE, suggested this bias was minimal. Furthermore, validation using cause-specific metrics that account for the competing risk of death confirmed the transformer’s robustness by mitigating potential overestimation of event probabilities.

Key areas for future work include translating the model into clinical practice and refining its predictive capabilities. We have shown that the transformer model is generalizable by successfully validating it on two different healthcare systems with differing cohort characteristics. We have additionally outlined its possible integration into existing EHR-enabled screening infrastructures such as PASI within the VA Healthcare system, in ***Integration in clinical workflows***. Though a detailed specification of clinical implementations is outside the scope of this study, the transformer in its current form can be integrated into such existing systems as a potential screening tool with minimal operational overhead. Future implementation research will evaluate workload and resource implications under specific deployment models, including centralized vs decentralized workflows and staffing requirements. Key methodological components of these models will include process mapping, scenario-based workload modeling, and economic evaluation. Future work should also address the practical design of clinical workflows and the infrastructure required to translate high-risk predictions into timely interventions, such as targeted anticoagulant use. Acting on predictions to avert imminent VTE requires both timely model execution and a clinical system, potentially adapted from a platform like PASI, that can respond quickly. Developing, piloting, and evaluating this infrastructure, including optimization of prediction time-windows^54^, are critical next steps for development. These analyses are intentionally deferred to maintain focus on model development. In parallel, ethical considerations associated with continued surveillance and patient autonomy warrant further examination.

The proposed model may also be improved by incorporating additional data sources, such as imaging results and unstructured clinical notes, or trained across differing health systems via transfer learning to better account for cohort-specific disparities. Vital indicators like BMI, though treated as a static categorical baseline covariate in this implementation, may be treated as time-varying EHR features in future work and integrated into a common vocabulary with phecodes and labs. Finally, the strong performance in certain patient subgroups merits further investigation to better understand the specific clinical drivers of risk in these distinct populations. Such directions may be explored by inferential analysis on additional external datasets.

In summary, we developed a transformer model that uses patient diagnostic and laboratory history from EHRs to predict VTE risk in cancer patients for up to one year following systematic treatment. The model is uniquely suited for dynamic risk assessment, as its performance improves deeper into a patient’s treatment history. This dynamism in short-term predictions gives the proposed transformer model a distinct role in informing clinical decisions, particularly for patients who have passed their treatment initiation more than six months.

## Methods

### Cohort and outcome definition

Data for this study were derived from the VA Corporate Data Warehouse (CDW), which contains comprehensive electronic health record (EHR) data from the VA’s 171 medical centers and 1113 outpatient clinics nationwide^55^, and the national VA Cancer Registry (VACR), which contains highly accurate, manually curated data on VA patients with cancer^56^. Records from January 2000 to June 2023 were used.

The primary derivation cohort included patients from the VACR who initiated systemic cancer therapy between 2011 and 2020. A separate temporal validation cohort was constructed for patients who began treatment between January 2021 and April 2022. Key exclusion criteria included not being a primary user of the VA healthcare system, not receiving systemic therapy within one year of diagnosis, and having a recent history of VTE or therapeutic anticoagulation use. The full patient inclusion and exclusion flowchart is outlined in Fig. S.I.1.

The primary outcome was overall VTE, defined as radiologically confirmed symptomatic or incidental pulmonary embolism (PE), proximal or distal lower extremity deep vein thrombosis (LE-DVT), or upper extremity DVT (UE-DVT)^20^. VTE events were ascertained using a previously validated algorithm with 96% recall and 98% precision, which leverages both structured data (diagnosis codes, medications) and unstructured data (natural language processing of radiology impressions)^57^.

Cohort and outcome definitions, input feature extraction, and data filtering for the HHS external validation set were done in the same way as for the VA. Details on derivations of both HHS and VA datasets, including patient linkage, harmonization, and exclusion, can be found in Li et al. (2021)^20^.

### Input feature extraction

Model inputs included both longitudinal (time-varying) and static (time-invariant) features derived from the EHR. Longitudinal features included time-stamped diagnostic codes from inpatient and outpatient encounters mapped to Phecodes from the *PheWas* repository. Phecodes are manually curated groups of ICD codes designed to capture clinically meaningful summary concepts and to create more concise representations by consolidating disease phenotypes into 1862 categories^37,38^. Missing or mismatched phecodes are mapped by default to the *unknown* category. Additional longitudinal features included binned laboratory values from complete blood cell count (CBC) and complete metabolic panel (CMP) tests, which are known to be associated with VTE^20,58,59^. The full list of labs is found in Fig. 2, and detailed information can be found in **Supplementary V.A**. Each time-stamped laboratory value was binned as *normal* if it is within the normal range, *low* if below, *high* if above, or *unknown presence* if it is missing or an outlier. Any longitudinal features recorded more than five years prior to the treatment index date were excluded. For model input, each longitudinal event was one-hot encoded and mapped to a low-dimensional vector.

Static features were derived from demographic information. Sex (male/female) was used as a binary covariate. BMI was binned into 4 categories (up to 4: Class II obesity) – time varying fluctuations were captured by phecodes. Race and ethnicity were binned into six categories as in Table 1, including *unknown*. These static features were incorporated as neurons in the model’s final linear layers.

### Data filtering and quality control

The primary cohort was randomly split into a ratio of 60:20:20 for the training, development, and test sets (Table S.I.1**)**. For all training, development, test, and temporal validation sets, we truncated input data to exclude any events occurring within 90 days prior to the cancer diagnosis date (the start of the trajectory in the main analysis). After splitting and truncation, we required that each patient have no fewer than 5 input events in all sets. Additionally, to control for systematic delays in treatment attributable to COVID-19-related externalities from 2021–2022 in the internal temporal validation set^60–62^, we imposed a simple debiasing filter by removing patients without timely treatment (time to treatment > 4 months) to better match those in the development cohort (Figure S.IV.4**)**.

### Model training and development

Model training was performed exclusively on the training set using the Adaptive Moment Estimation (ADAM) as the descent algorithm, while the development set was used to evaluate performance and to serve as benchmarks for model selection. Each patient is split and resampled to up to 4 trajectories, with criteria described in ***Patient EHR trajectories***. Trajectories were sampled in small batches for efficient computation. Given the rarity of VTE events, class balancing was implemented in the training set to maintain roughly equal proportions of positive and negative cases within each batch.

Hyperparameters, including transformer layers, heads, embedding dimension, and trajectory length, were adjusted to refine and develop the transformer. Ablated models without phecode or lab inputs, static covariates, or embedding anchor times were trained to assess the optimal combinations of input data types. Each model was trained for a total of 30 epochs with an initial learning parameter of 0.00125 and a weight decay of 0.3.

### Positional encoding

The transformer uses positional encoding to incorporate temporal information into the model, building on prior approaches used in cancer prediction^29,63^. For each patient, the timestamps associated with phecode and laboratory tokens are encoded using positional embeddings. This allows the model to capture the relative timing of clinical events, enabling dynamic and temporally-aware prediction of VTE risk over the patient’s clinical trajectory.

The novel aspect of our implementation is the use of differing anchor times in positional encoding, which enables the transformer to model temporal patterns in two distinct ways. (1) *End-of-trajectory anchor times* allow the model to differentiate between identical input events occurring at different points along the patient timeline. (2) *Diagnosis and treatment index anchor times* enable the model to weigh input events based on their temporal proximity to clinically meaningful reference points. In both approaches, events that occur closer to the anchor time are given greater emphasis.

We first define the positional encoding mechanism in general terms. Consider a trajectory ${\boldsymbol{\delta }}$ comprised of *N* events, with each input event indexed as i with timestamp at ${\delta }^{i}$, which is tokenized by ${v}^{i}$ with vocabulary size ${\mathscr{V}}$. ${\boldsymbol{\delta }}$ is mapped to a low-dimensional continuous feature vector with dimension *d*, forming an input feature matrix ${\bf{X}}\epsilon N\times d{\boldsymbol{.}}$ Embeddings are positionally encoded to account for the temporal order of events within a trajectory and allow the model to handle irregularly spaced events. We generate positional time embeddings using a set of multipliers $M=2\pi /{\rm{linspace}}({start},{end},d)$ $\epsilon {{\bf{R}}}^{d}$, where *start* and *end* denote the minimum and maximum values for the embedding range, and linspace posits *d* linearly spaced points between *start* and *end*. The time difference between event *i* and a given reference time R is positionally encoded by a cosine function:

1 $$P\left(i,R\right)=\cos \left(\left|{\delta }^{i}-R\right|\cdot M\right)\epsilon {{\bf{R}}}^{N\times d}.$$

Let Θ denote the overall set of trainable parameters. For arbitrary vector positional encoding ${\boldsymbol{P}}$ with corresponding learnable linear layers ${{\bf{W}}}_{{scale},{\boldsymbol{P}}}\left(\Theta \right),{{\bf{W}}}_{{bias},{\boldsymbol{P}}}(\Theta )$ parameterized by weights from Θ, we define scale and bias components of embedding ${{\bf{E}}}_{{scale},{\boldsymbol{P}}}(\Theta )={{\bf{W}}}_{{scale},{\boldsymbol{P}}}(\Theta )\cdot {\boldsymbol{P}}$, ${{\bf{E}}}_{{bias},{\boldsymbol{P}}}(\Theta )={{\bf{W}}}_{{bias},{\boldsymbol{P}}}(\Theta )\cdot {\boldsymbol{P}}$. Let ⨁ denote the scaling-and-biasing operation of the positional encoding with respect to input feature matrix **X,**

2 $${{\boldsymbol{P}}}_{\Theta }\oplus {\bf{X}}={{\bf{E}}}_{scale,{\boldsymbol{P}}}(\Theta )\odot {\bf{X}}+{{\bf{E}}}_{bias,{\boldsymbol{P}}}(\Theta )$$

where ⊙ denotes element-wise multiplication.

### Transformer model architecture

Now we specify the transformer model tailored for the prediction of VTE risk. Consider input trajectories with associated positional encodings specified for each patient j=1,…,n. Each patient has a varying-length trajectory ${{\boldsymbol{\delta }}}_{j}$ with ${N}_{j}$ events of merged phecode and lab records, tokenized by ${{\boldsymbol{v}}}_{j}\epsilon {N}_{j}$ with vocabulary size ${\mathscr{V}}$ = 1934 (1862 phecodes and 72 lab combinations). Each ${{\boldsymbol{\delta }}}_{j}$ is mapped to a lower-dimensional continuous input feature matrix ${{\bf{X}}}_{j}\epsilon {N}_{j}\times d$. The primary embedding uses the *end* time (last observation of trajectory indexed as *t* = I, II, III, or IV for patient j) as the reference time: ${P}_{i,j,t}^{{End}}={P}_{\left(j\right)}\left(i,{End}(j,t)\right)$, dependent on trajectory index *t*. Here, the transformer distinguishes between the same input at different times relative to each trajectory's end time. Additional embeddings (Eq. 1) about anchor dates specific to patient *j* are calculated:

(1) treatment index date ${{Tr}}_{(j)}:{P}_{i,j}^{{Tr}}={P}_{j}(i,{{Tr}}_{(j)}),$

(2) diagnosis date $${{Dx}}_{\left(j\right)}:{P}_{i,j}^{{Dx}}={P}_{j}(i,{{Dx}}_{(j)})$$,

(3) age at end time (of trajectory *t*) using date of birth ${{DOB}}_{(j)}$: ${A}_{j,t}={P}_{j}({{DOB}}_{(j)},{End}(j,t)).$

Vector embeddings ${A}_{j,t},{{\boldsymbol{P}}}_{j}^{{Tr}},{{\boldsymbol{P}}}_{j}^{{Dx}},{{\boldsymbol{P}}}_{j,t}^{{End}}$ are integrated with the patient-specific input feature matrix ${{\bf{X}}}_{j}$. Let Θ represent the model parameters specific to the transformer. For each trajectory *t*, embedded sequences are combined (Eq. 2) to form a composite:

$${{\bf{E}}}_{t}(\Theta ,{{\bf{X}}}_{j})={\left({A}_{j,t}\oplus {{\boldsymbol{P}}}_{j}^{{Tr}}\oplus {{\boldsymbol{P}}}_{j}^{{Dx}}\,{\oplus {\boldsymbol{P}}}_{j,t}^{{End}}\,\right)}_{\Theta }\,\oplus {{\bf{X}}}_{j}{\boldsymbol{.}}$$

${{\bf{E}}}_{t}(\Theta ,\,{{\bf{X}}}_{j})$ is then passed through a multi-head attention (*MHA*) layer. Multi-head attention extends the standard attention mechanism^24^ ${Attention}(Q,K,V)$ by splitting it into *H* heads. Each ${{head}}_{h}=\,{Attention}\left({Q}_{h},{K}_{h},{V}_{h}\right)$ are computed across h=1,…,H, where Queries ${Q}_{h}$, Keys ${K}_{h}$, and Values ${V}_{h}$, are obtained from learned linear layer projections. The full ${MHA}\left(Q,K,V\right)$ is obtained by concatenating these heads and multiplying by an additional linear layer ${{\bf{W}}}_{0}\left(\Theta \right)\epsilon {{\bf{R}}}^{H\times d}$, then layer-normalized and fed to a fully connected feedforward neural network (FFN), followed by Linear, Pooled, and SoftMax layers (Fig. 1). Prediction ${\hat{p}\,}_{\Theta ,t+1}{({\bf{X}}}_{j,{t}})$ at quarter *t* + *1* is then derived from a composition of all such layers, written as follows:

$${\hat{p}\,}_{\Theta ,t+1}{({\bf{X}}}_{j,t})\propto {\rm{SoftMax}}\circ {\rm{Pooled}}\circ {\rm{Linear}}\circ {\rm{FFN}}\circ {\rm{LayerNorm}}\circ {MHA}\left(Q,K,V\right)\circ {{\bf{E}}}_{t}\left(\Theta ,{{\bf{X}}}_{j}\right){\boldsymbol{.}}$$

This transformer is then trained to minimize binary cross-entropy loss using ADAM:

$$\mathop{\min }\limits_{\Theta }\mathop{\sum }\limits_{j,\,t}-{\bf{1}}\left({y}_{j,t+1}\right)\left({y}_{j,t+1}\log \left({\hat{p}}_{\Theta ,t+1}\left({{\bf{X}}}_{j,t}\right)\right)+{(1-y}_{j,t+1})\log \left(1-{\hat{p}}_{\Theta ,t+1}\left({{\bf{X}}}_{j,t}\right)\right)-\lambda |\Theta |\right){\boldsymbol{.}}$$

For patient j = 1,…,*n*, each $t+1=\{{Q}_{1},{Q}_{2},{Q}_{3},{Q}_{4}\}$ represents each of the four quarters looking *forward* from trajectory index *t*, outcome ${y}_{j,t+1}$ is 1 for VTE or 0 if no outcome. The indicator ${\boldsymbol{1}}({y}_{j,t+1})$ is 0 if the patient has had a previous event at an earlier quarter, died, or dropped out. ${{\bf{X}}}_{j,t}$ represents the input trajectory of patient *j* at interval *t*, and ${\hat{p}}_{\Theta ,t+1}$ is the estimated predicted risk at quarter *t* + 1 with respect to Θ and ${{\bf{X}}}_{j,t}$. λ represents the *L2* penalty parameter.

### Model calibration

We recalibrated the prediction probabilities in the test and temporal validation sets using a three-step procedure. First, we modeled positive case counts of the case-balanced training set with a normal distribution and negative cases with a zero-inflated Poisson distribution. Second, we applied the case-balancing approach of the training set to simulate case-balancing in the development set, which served as the calibration set. Parameters of the Poisson and normal distributions were tuned to ensure the predicted probabilities were well-calibrated against observed frequencies. Finally, cubic spline models were trained on the rebalanced calibration set, weighted by the simulated sampling frequencies. Spline parameters (number of knots, smoothing penalty) were optimized using the calibration set^64,65^.

### Model evaluation

The Area Under the Receiver Operating Characteristic Curve (AUROC, or AUC) served as the primary metric for training, evaluation, and comparison. As in model training, each patient in the test and validation sets is split and resampled into up to four trajectories to predict Q1-Q4. AUCs for each quarterly prediction in the test set were evaluated separately. We compared AUCs for sensitivity and subgroup analyses, as well as internal (temporal) and external validations.

Confusion matrices and associated metrics were used in addition to AUCs for model evaluation after recalibration. Calibration was evaluated using reliability plots in the training, test, and temporal validation sets. Confusion matrices for recalibrated Q1-Q4 predictions were constructed using the empirical (crude) incidence rates from the calibration set as the cutoff thresholds for positive classification (3.4%, 1.9%, 1.1%, and 0.9% for Q1-Q4). Recall, which indicates how many true positive cases are correctly identified, and precision, the ratio of true positive predictions to the total number of positive predictions, were evaluated after risk classification.

Test set predictions were verified to be minimally dependent of time-to-prediction. Time-stratified test-set performance was also evaluated (**Supplementary IV.A)**. Performance on the hold-out temporal validation set was used as additional internal validation to demonstrate the model’s cross-applicability when applied to future timepoints.

### Competing risks analysis

We accounted for the competing risk of death in two ways. Firstly, we calculated CS-AUCs^66^, which quantified discrimination among individuals at risk for a specific event (e.g., VTE) who have not experienced any event (VTE, dropout, or death) by time *t*. We used IPCW^67^ to properly adjust for patients who died or were censored before *t*. The CS-AUC was estimated using Fine-Gray (FG) subdistribution hazard models, using the transformer’s logit-transformed outputs as the sole predictor^68,69^. The outcome of the FG model was defined using a multi-state endpoint that included: time to VTE (encoded as 1: event of interest), time to death not attributed to VTE (2: competing risk), time to dropout or end of prediction window (0: censoring).

Additionally, we augmented the recalibration model (***Model calibration)*** to incorporate CIFs to account for the competing risk of death^70,71^. The spline fitted from the calibration set was used as the sole predictor in the FG model towards the same time-to-event outcome as described above (1: VTE, 2: death, otherwise 0). CIFs for VTE were then estimated from this model at 15-day time bins across the prediction quarter and matched to each patient’s nearest (left-truncated) time of event or censoring to generate recalibrated calibration curves.

### Model attribution

To identify phecodes and laboratory tests most strongly associated with VTE, we estimated attribution weights using Layer Integrated Gradients (LIG) for predictions across Q1–Q4^72^. This method quantifies the contribution of each input feature by integrating gradients along the path from a baseline to the actual input. For each prediction interval, attribution scores reflect the degree to which specific features are correlated with patients who develop VTE during that quarter. Token-level attributions were obtained by summing contributions across all embedding dimensions and aggregating across the full set of patient trajectories.

### Model comparison

The EHR-CAT risk assessment score^20^ and Khorana score^13^ were evaluated as comparator methods. The EHR-CAT score is defined using 11 covariates, including BMI, race, cancer stage, and CBC lab values. The model was replicated with input and prediction intervals that match the setting of the transformer^36^. Confusion matrices for EHR-CAT and Khorana scores were constructed with a classification threshold of RAM ≥ 3. Comparisons of associated efficiency metrics NNE^44^ and NNS-V^45^ were calculated assuming thromboprophylaxis with DOAC^9^ (Table S.IX.1**)**. Kaplan-Meier curves were calculated for additional comparison (Fig. S.VIII).

### External validation on the Harris Health System

We applied the transformer model developed on the VA cohort to the HHS cohort to predict VTE risk. Longitudinal features (1862 phecodes and 72 lab values), static features, and anchor times were coded in the same way as for VA. Model evaluation in the HHS cohort was conducted using the same measures and statistical analysis as for the evaluation in the VA cohort.

### Integration in clinical workflows

Risk assessment models (RAMs) have been successfully integrated into clinical workflows for screening conditions such as lung cancer^73,74^ and demonstrate the feasibility of this approach for VTE risk prediction. The Prediction Augmented Screening Initiative (PASI) at the VA healthcare system provides a model for rapid deployment of such tools^75^. PASI is an EHR-enabled recommender system for clinicians that pulls patient data nightly and displays results through a user interface, ranking patients by predicted risk. Following PASI’s successful pilot with subsequent expansion to 28 VA sites, we propose a similar infrastructure for VTE screening in patients undergoing systemic cancer treatment. Using the ENTHRALL platform, which is designed to support multiple clinical applications, this system can integrate the transformer model as an automated screening tool for VTE with highly streamlined workflows and regularized labor-time estimates.

We outline a clinical workflow for this prediction-augmented VTE screening system. With patient consent, the system continuously updates diagnoses and laboratory records through data pulls. The screening workflow consists of three stages:

1. *Initial triage (1–3* *minutes)* An automated triage system flags patients when newly ingested medical records are classified by the transformer as high risk. As with PASI, ineligible patients are automatically excluded, and remaining recommendations are grouped by risk level (e.g., high risk, borderline), giving clinicians immediate visual prioritization.
2. *Chart review (3–20* *minutes)* The clinical reviewer assesses the prediction and, if complex (~20%), escalates it to a physician for further assessment. Along with each recommendation, the system provides relevant patient data, including comorbid conditions, exposure history, and extracted clinical notes, supporting informed clinical judgment.
3. *Clinical decision (1-2* *minutes)* Prophylactic anticoagulation is prescribed after assessing bleeding risk, with additional monitoring by standard protocol^76^ and/or tests such as ultrasound for asymptomatic DVT. VTE risk is reassessed quarterly.

This prediction-augmented infrastructure-supported workflow has a median screening time of 10 minutes (IQR [5,25]:) depending on case complexity. A detailed diagram is shown in Fig. S.IX.1.

To quantify the operational workload of the proposed screening process, we calculated the *labor-time per VTE detected* (LTV) using the formula

$${\rm{LTV}}=t({\rm{VTE}})\times {\rm{NNE}}$$

where *t* (VTE) is the time required per true VTE detected, and NNE is defined as 1/precision^44^. We assess the LTV of the transformer model based on the approximated times to quantify real-world clinical burden.

### Ethics statement

The study was approved by the Research and Development Committee and Institutional Review Board of the VA Boston Healthcare System and the Institutional Review Board of Baylor College of Medicine. It received a waiver of informed consent because the study presented minimal risk and could not practicably be conducted without a waiver.

## Supplementary information

Supplementary information.

## Data Availability

Analysis of VA data was conducted under a waiver of informed consent with approval from the VA Boston Healthcare System Institutional Review Board. All VA data used in this study are available to any investigator upon relevant approvals through the VA Informatics and Computing Infrastructure (VINCI) (contact: VINCI@va.gov). Those who wish to access the data and to use them for research purposes will be required to meet research credentialing requirements as outlined by the VA Office of Research and Development, which is expected to be processed within at least 6 months. HHS data used for external validation were collected under a waiver of informed consent from an IRB from Baylor College of Medicine; data are available upon relevant approval following rigorous review of all protocols by its Research and Sponsored Programs (research@harrishealth.org). Code for the model is accessible at https://github.com/thunder001/VTERisk/. All analyses carried out in this study were performed using Python version 3.11.7. The Python package torch version 2.2.0 was used for developing the machine learning models, and LIG was implemented using the Python package *Captum* version 0.7.0. Visualizations were performed in R base plot, package *CalibrationCurves*, and *ggplot2* (version 3.5.2). R packages *cmprsk* and *riskRegression* were used for competing risks analysis, and *smooth*.*spline* was used for recalibration models.
