# Supplementary material for: A deep learning model to dynamically predict cancer-associated thromboembolism in large-scale healthcare systems
Source: NPJ Digit Med. 2026 May 18;9:602. doi: 10.1038/s41746-026-02730-2 (PMC13443184; doi:10.1038/s41746-026-02730-2)
Supplement: Supplementary file 1 [file 41746_2026_2730_MOESM1_ESM.pdf]

Supplemental Material

He, et al. *A deep learning model to dynamically predict cancer associated thromboembolism in large-scale healthcare systems*

List of Supplements

Supplement I. Cohort Details
Supplement I.A Derivation and details of primary Cohort (VA; treatment index dates 2011-2020)
Supplement I.B Descriptive table of internal validation Cohort (VA; treatment index dates 2021-2022)
Supplement I.C Descriptive table of external validation Cohort (HHS; treatment index dates 2011-2020)

Supplement II. Sensitivity analyses
Supplement II.A Model calibration
Supplement II.B Sensitivity analyses

Supplement III. Subgroup analysis
Supplement III.A Subgroup analysis in primary cohort (VA)
Supplement III.B Subgroup analysis in external validation cohort (HHS)

Supplement IV. Temporal diagnostics
Supplement IV.A Cross-sectional diagnostics
Supplement IV.B Temporal validation diagnostics

Supplement V. Details of input features

Supplement VI. Attribution analysis

Supplement VII. Competing risks analysis

Supplement VIII. Kaplan Meier curves

Supplement IX. Efficiency metrics and operational workload quantification

List of Supplementary Figures and Tables

Figure S.I.1. Patient and cohort inclusion and exclusion criteria.
Table S.I.1. VTE Incidence rates by training, development, and test sets. Control (non-VTE) subjects are re-sampled at each timepoint until dropout or death.

Table S.I.2. Patient characteristics of temporal validation set (2021-2022)
Table S.I.3. Patient characteristics of HHS external validation set (2011-2020)

Table S.II.1. Optimal hyperparameters for the transformer model
Table S.II.2. Ablation Analysis with fixed trajectory settings (Max length = 800, 90 ± 10 days before diagnosis, 1 layer, 8 heads, 400 pad size) as the main result.
Table S.II.3. Sensitivity Analysis with varying start-times, maximum # events, and pad sizes.
Table S.II.4. Sensitivity Analysis with varying layers, heads, minimum lengths
Table S.II.5. Sensitivity analyses with varying start times based on patients' time-to-treatment from diagnosis (TTD). Longer TTD corresponds with shorter the look-back windows.
Table S.II.6. Spline Calibration Parameters

Figure S.II.1. Calibration plot for the training set with artificial class balancing for Q1-Q4
Figure S.II.2. Calibration plots for test set predictions from the transformer (TF) and the EHR-CAT score for Q1-Q4.
Figure S.II.3. Calibration plot for the temporal validation set for Q1-Q4.

Figure S.II.4. Confusion matrix/calibration plots for alternate spline parameters
Figure S.II.5. Confusion matrix/calibration plots for Phecode model
Table S.II.7. Q1 model sensitivity comparison

Table S.III.1. Model Performance stratified by cancer type, cancer stage, and treatment across cancer type, early-intermediate cancer stages, and endocrine / immune therapy were grouped due to small sample size.
Table S.III.2. Demographic subgroup analysis**.** Performance of the proposed model stratified by age, race/ethnicity, and sex subpopulations.
Table S.III.3. Demographic subgroup analysis for HHS external validation cohort stratified by age, race/ethnicity, and sex subpopulations matching that of the primary VA test set.
Table S.III.4. Breast cancer subgroup performance in HHS validation cohort

Figure S.IV.1. *(Top)* Predicted (raw) probabilities of VTE patients (all sets) compared to day of occurrence post-treatment index date (stratified by in lookforward window). (*Bottom*) Histograms of days-to-VTE for each 3-month prediction window, separated by positive and negative predictions (post-calibration and classification into pred+/pred-).
Figure S.IV.2. VTE incidence rate (all quarters combined) for both the primary cohort and the temporal validation set (split indicated by the black dashed line). The grey dotted line indicates the onset of the COVID-19 pandemic starting in 2020.
Figure S.IV.3. Frequency of index dates (start times of systemic cancer treatment) for both the primary cohort and the temporal validation set (split indicated by the dashed line).
Figure S.IV.4. Mean times to treatment from diagnosis (TTD) for the test and validation sets at given prediction quarter., for all patients and patients who develop VTE at the given quarter. Effects of the 120-day TTD filter are also shown.

Table S.IV.1. Cross- Sectional temporal validation for test set (2011-2020): characteristics and performance results of filtering time-to-treatment-from-diagnosis (TTD) by 120 days.
Table S.IV.2. Cross- Sectional temporal validation for test set (2020)

Table S.IV.3. Cross- Sectional temporal validation for test set (2011-2019)
Table S.IV.4. Validation set performance on total temporal validation cohort, without filtering by time-to-treatment, from Jan. 2021-Apr. 2022.

Table S.V.1. Trajectory characteristics of full phecode and lab features for the full dataset in the primary cohort (5 years before and 2 years after index date).
Table S.V.2. Laboratory test Characteristics of primary cohort
Figure S.V.1. Relative frequencies of phecode categories for the full dataset in the primary cohort.

Figure S.VI.1. Attribution analysis: Attribution weights for the TF model at each prediction interval calculated from layer integrated gradients. Attributions were calculated for merged trajectories of disease and lab tokens. Frequencies with counts of at least 10 from layer integrated gradients were retained for each quarter.
Figure S.VI.2. Trends of top attributions Top attribution weights plotted over time, split into those that are increasing and decreasing across the four intervals.

Figure S.VII.1. Cause-specific AUC (CS-AUC) for VTE risk for each prediction window in the test set, accounting for the competing risk of death (not attributable to VTE).
Figure S.VII.2. Calibration plots for recalibrated predictions using CIFs for VTE as observed probability bins.

Figure S.VIII. Kaplan-Meier curves for TF and EHR-CAT model. Cumulative incidence plots, split by VTE and non-VTE, across time for the four prediction intervals for the TF and EHR-CAT models. VTE incidence dates, dates of dropout, or the maximum time-window censor (90 days) are used as event dates.

Table S.IX.1. Precision, recall, NNE (number needed to evaluate among positive predictions) and NNS (number needed to screen to prevent one positive case of VTE) **(**assuming absolute risk reduction (ARR) of 5% for on-treatment DOAC use for all quarters) for transformer, EHR-CAT, and Khorana Score (KS) models.

Figure S.IX.1. Clinical screening workflow diagram within a prediction-augmented infrastructure. Modeled after PASI, the system ingests patient data nightly through extract, transform, and load (ETL) processes, applies NLP algorithms to extract clinical variables from unstructured notes, generates risk scores using the transformer model, and displays ranked patient lists through a user interface. Clinicians review flagged patients, confirm eligibility, and initiate appropriate follow-up with standardized documentation templates, enabling consistent workflows and measurable labor-time estimates across sites.

Table S.IX.2. Operational workload analysis for the transformer model within prediction-augmented clinical workflow assuming 10 (IQR: 5-25) minutes per true positive case of VTE.

Supplement I: Cohort details
Supplement I.A: Details of primary cohort (VA with treatment index dates 2011-2020)

Figure S.I.1: Patient and cohort inclusion and exclusion criteria. Details of derivation are shown for both the primary and temporal validation cohorts. An "EHR event" indicates a time-stamped lab record or phecode. "Trajectory start" indicates 90 days before cancer diagnosis date.

**Full Cohort:** VA Cancer Registry (VACR) + CDW 2006-2022	
Exclude if nonanalytic cases (cancer diagnosis was not from VA hospital)	*n = 808,926*
Exclude if benign histology	*n = 532,341*
Exclude if not primary VA user (1 or more workload encounter or event in CDW in the past 2 years, with more than 30-day follow-up)	*n = 459,496*
Exclude if no systemic or oral anticancer therapy within 1 year after diagnosis	*n = 163,338*
Exclude if received anticoagulation therapy within 30 days before treatment index date	*n = 153,540*
Exclude if missing cancer group, height, weight, demographic variables or age <18	*n = 125,854*
Exclude if recent history of acute VTE within 6 months prior to treatment index date	*n = 123,648*
Exclude if systemic treatment index date is before 2011	*n = 84,922*
Exclude if systemic treatment index date is before after 2020	*n = 80,808*

**Primary Cohort**	Random Split	Exclude if < 5 EHR events after trajectory start		
Training Set	60%	*n = 48,484*	*n = 48,414*	*Model development*
Development Set	20%	*n = 16,162*	*n = 16,094*	*Model development*
Test Set	20%	*n = 16,162*	*n = 16,132*	*Model evaluation*

**Temporal Validation Cohort** (Treatment index date between 2021-2022)		
Exclude if systemic treatment index date is before 2021	*n = 4,114*	*Model evaluation*
Exclude if < 5 EHR events after trajectory start	*n = 4,098*	*Model evaluation*
Exclude if did not receive timely treatment (< 4 months from diagnosis to treatment index date)	*n = 3,303*	*Model evaluation*

Table S.I.1: VTE Incidence rates by training, development, and test sets. Control (non-VTE) subjects are re-sampled at each timepoint until dropout or death.

**Prediction Quarter** (months after index date)	**TRAIN**		**DEVELOPMENT**		**TEST**	
	**# Patients**	**# VTE (%)**	**# Patients**	**# VTE (%)**	**# Patients**	**# VTE (%)**
**Total**	**48414**	**2883 (6.0%)**	**16094**	**1042 (6.5%)**	**16132**	**954 (5.9%)**
Q1: 0-3 Months	48414	1546 (3.2 %)	16094	541 (3.4%)	16132	480 (3.0%)
Q2: 3-6 Months	42588	678 (1.6 %)	14170	274 (1.9%)	14226	220 (1.5%)
Q3: 6-9 Months	38349	359 (0.9%)	12735	137 (1.1%)	12829	153 (1.2%)
Q4: 9-12 Months	34122	294 (0.9%)	11281	90 (0.8%)	11681	101 (0.9%)

Supplement I.B: Cohort details of internal temporal validation set (treatment index dates 2021-2022)

**Table S.I.2:** Patient characteristics of temporal validation set (2021-2022)	**Did not experience VTE** within 1 year of treatment	**Experienced VTE** within 1 year of treatment
**Sample Size** (total = 3303)	3090 (93.6%)	213 (6.4% prevalence)
Q1 (0-3 months, total = 3303), n (%)	3171 (96.0%)	132 (4.0% incidence)
Q2 (3-6 months, total = 2880)	2832 (98.7%)	48 (1.7% incidence)
Q3 (6-9 months, total = 2639)	2623 (99.4%)	16 (0.6% incidence)
Q4 (9-12 months, total = 2453)	2436 (99.3%)	17 (0.7% incidence)
**Demographics**		
**Age at treatment index date,** median (IQR)	71.4 [64.0, 75.3]	70 [63, 74.1]
**Sex,** n (%) Female	229 (7.4)	12 (5.6)
**Race**, n (%)		
White	2252 (72.9)	148 (69.5)
Black or African American	686 (22.2)	56 (26.3)
American Indian or Alaskan Native	20 (0.6)	3 (1.4)
Asian, N. Hawaiian, Pacific Islander	26 (0.8)	1 (0.5)
Unknown	106 (3.4)	5 (2.3)
**Ethnicity** (Hispanic/Latino), n (%)	63 (2.1)	4 (1.6)
**BMI Category**		
Underweight or healthy weight (0- 24.9)	764 (24.7)	48 (22.5)
Overweight (25-29.9)	1045 (33.8)	73 (34.3)
Obese (30-34.9)	765 (24.8)	51 (23.9)
Obese II (≥ 35)	516 (16.7)	41 (19.2)
**Primary Cancer Type (**Most common among primary cohort**)**		
Lung and Bronchus	447 (14.5%)	38 (15.5%)
Prostate	468 (15.1%)	9 (3.3%)
Liver	106 (3.4%)	5 (1.9%)
Urinary Bladder	115 (3.7%)	18 (6.6%)
Multiple Myeloma	241 (7.8%)	18 (6.1%)
Esophagus	85 (2.8%)	6 (2.8%)
Pancreas	102 (3.3%)	21 (9.9%)
**Cancer Stage,** n (%)		
Early (0 or I)	872 (28.2%)	68 (31.9%)
Intermediate (II or III)	972 (31.5%)	65 (30.5%)
Advanced (IV)	1246 (40.3%)	80 (37.6%)
**Primary Treatment Type (not mutually exclusive)**, n (%)		
Chemotherapy	2055 (66.5%)	180 (84.5%)
Endocrine and Immunotherapy	790 (25.5%)	29 (11.7%)
Targeted Therapy	647 (20.9%)	40 (18.8%)
**Follow up times (days),** mean [IQR]		
Days to VTE from treatment		96 [28, 142]
Days to treatment from diagnosis (TDD)	79 [27, 100]	62 [25, 76]

Supplement I.C: Cohort details of external validation set (HHS; treatment index date 2011-2020)

**Table S.I.3:** Patient characteristics of HHS external validation set (2011-2020)	**Did not experience VTE** within 1 year of treatment	**Experienced VTE** within 1 year of treatment
**Sample Size** (total = 9752)	9031 (92.6%)	721 (7.4% prevalence)
Q1 (0-3 months, total = 9752), n (%)	9374 (96.1%)	378 (3.9% incidence)
Q2 (3-6 months, total = 8537)	8353 (97.8%)	184 (2.2% incidence)
Q3 (6-9 months, total = 7244)	7151 (98.7%)	93 (1.3% incidence)
Q4 (9-12 months, total = 5962)	5896 (98.9%)	66 (1.1% incidence)
**Demographics**		
**Age at treatment index date,** median (IQR)	55.1 [46.5, 62.0]	55.2 [46.0, 62.1]
**Sex,** n (%) Female	5159 (57.1%)	388 (53.8%)
**Race**, n (%)		
White	1339 (14.8%)	136 (18.9%)
Black or African American	2320 (25.7%)	249 (34.5%)
American Indian or Alaskan Native	12 (0.1%)	0 (0.0%)
Asian, N. Hawaiian, Pacific Islander	525 (5.8%)	13 (1.8%)
Unknown	75 (0.8%)	12 (1.7%)
**Ethnicity** (Hispanic/Latino), n (%)	4760 (52.7%)	311 (43.1%)
**BMI Category**, n (%)		
Underweight (0- 24.9)	421 (4.7%)	27 (3.7%)
Healthy weight (25-29.9)	2865 (31.7%)	249 (34.5%)
Overweight (30-34.9)	2863 (31.7%)	222 (30.8%)
Obese (≥ 35)	2882 (31.9%)	223 (30.9%)
**Primary Cancer Type** (most common among primary VA cohort), n (%)		
Lung and Bronchus	708 (7.8%)	101 (14.0%)
Prostate	549 (6.1%)	17 (2.4%)
Liver	252 (2.8%)	8 (1.1%)
Urinary Bladder	63 (0.7%)	19 (2.6%)
Multiple Myeloma	191 (2.1%)	9 (1.2%)
Esophagus	346 (3.8%)	47 (6.5%)
Pancreas	190 (2.1%)	31 (4.3%)
**Primary Treatment Type** (not mutually exclusive), n (%)		
Chemotherapy	6995 (77.5%)	643 (89.2%)
Endocrine therapy	168 (1.9%)	20 (2.8%)
Immunotherapy	1262 (14.0%)	27 (3.7%)
Targeted therapy	606 (6.7%)	31 (4.3%)
**Follow up times (days),** mean [IQR]		
Days to VTE from treatment		84 [35, 162]
Days to treatment from diagnosis (TDD)	59 [32, 101]	47 [23, 83]
**EHR trajectory features,** mean [IQR]		
# Input features up to treatment index date	101 [64, 164]	125 [79, 208]

Supplement II: Model details and sensitivity analyses

Table S.II.1 Optimal hyperparameters for the transformer model

**Parameter**	Setting **(**optimal in **bold)**
Number of hidden layers (L)	**1**, 2, 4
Number of heads (H)	4**, 8,** 16
Dimension of hidden layers (d)	32, 64, **128,** 256
Min length of trajectory	3, **5,** 10, 20
Dropout	**0**, 0.1, 0.2
Weight decay	0**, 0.3,** 0.5
Learning rate	0.0015**, 0.00125,** 0.001
Pad size	200, 300, **400**, 500
Maximum Length	200, 400, 500, **800**, 1000, 1200
Trajectory start time (days before diagnosis)	540, 360, 180, 120, **90**, 60, 0
Noise (days) about start time	30, 20, **10**, 0
Training batch size	128, **256**, 512
Development batch size	64, **128**, 256

Supplement II.A: Model calibration

Raw training set predictions from the raw algorithm output, where class balancing is pre-imposed. Predictions are slightly under-calibrated.

Figure S.II.1. Calibration plot for the training set with artificial class balancing

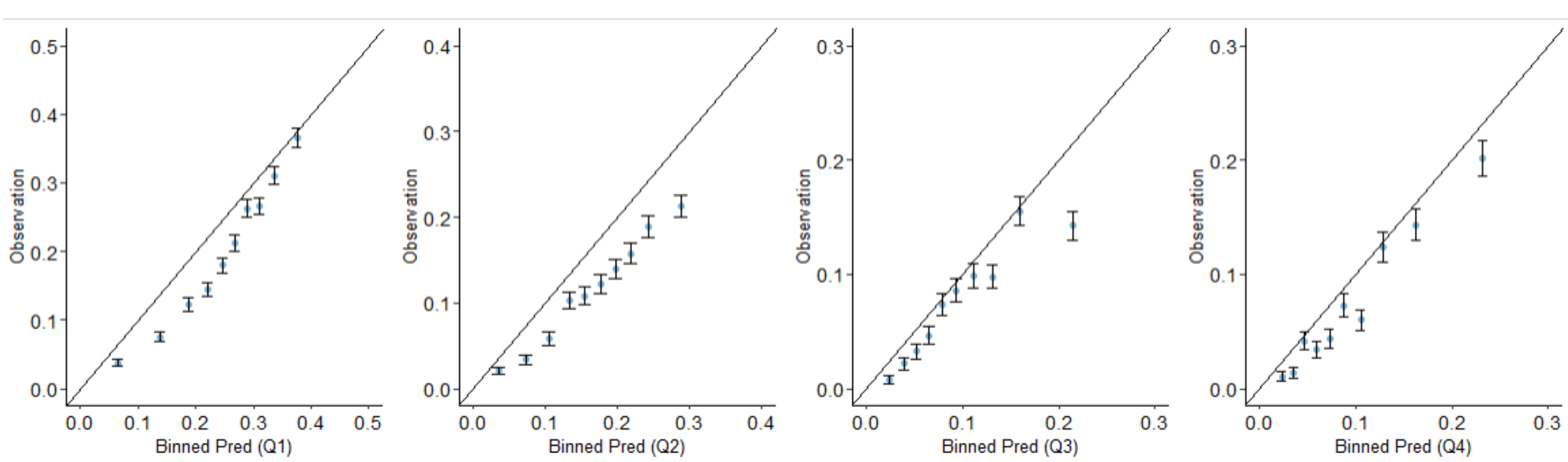

Figure S.II.2 Calibration Plots for test set predictions from the transformer and the EHR-CAT score across the four prediction intervals. Calibration plots show the decile-binned calibrated probabilities compared to the empirical rates of VTE incidence. The development set is used as the calibration set. For each interval, logit-transformed class-balanced raw prediction probabilities from the calibration set used to fit a cubic spline model and then the model is fitted onto logit-transformed raw prediction probabilities from the test set. *Number of knots* and *penalty* parameters are adjusted for each spline model to refine the test-set calibration. Raw probabilities for the TF model are extracted directly from the transformer, EHR-CAT probabilities are derived from the score divided by the maximum (5).

Transformer

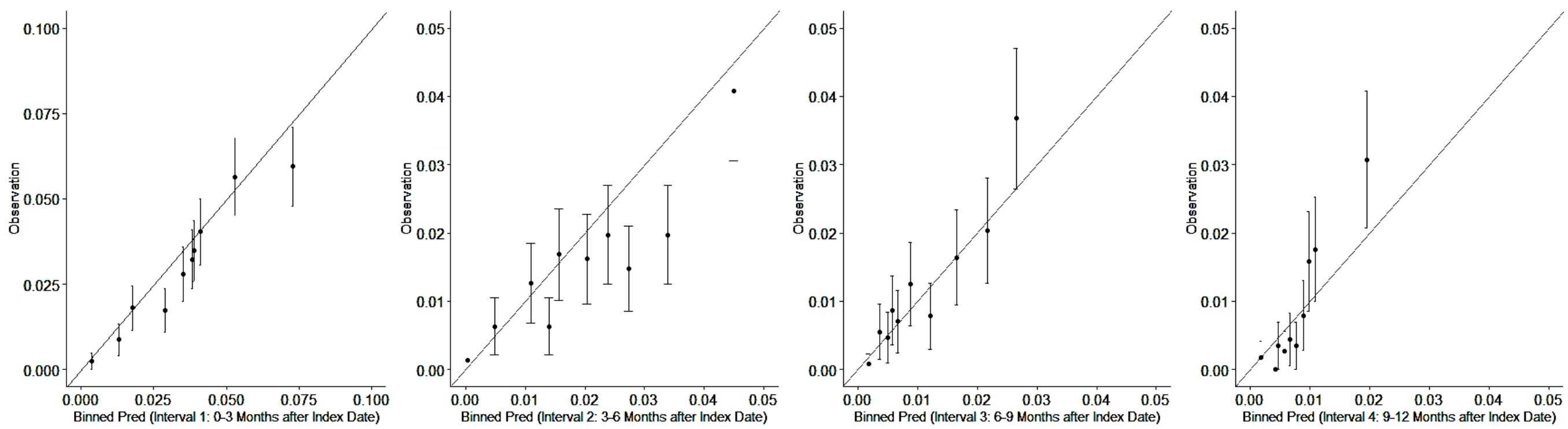

EHR-CAT

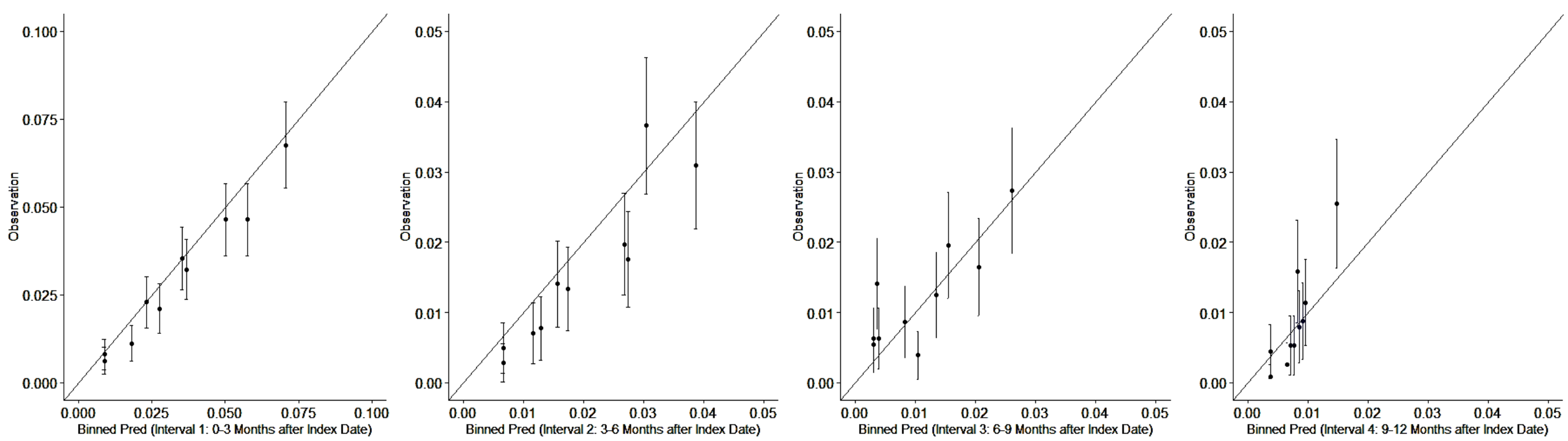

Figure S.II.3. Calibration plot for the temporal validation set after recalibration using the same spline calibration method as in the test set

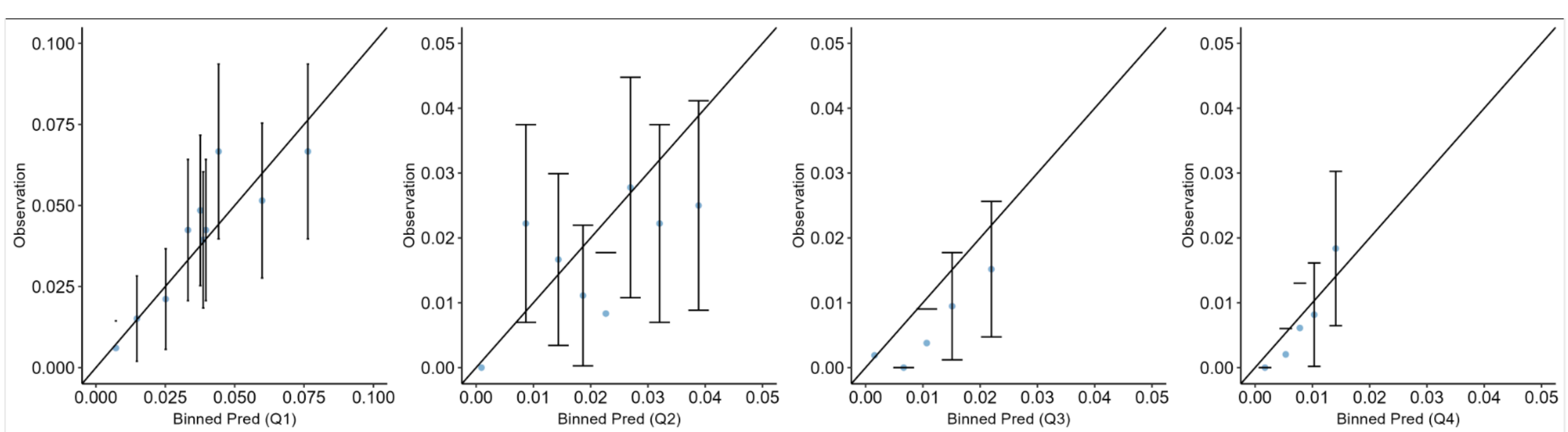

Supplement II.B: Sensitivity analyses

Table S.II.2: Ablation analysis with fixed trajectory settings (Max length = 800, 90 ± 10 days before diagnosis, 1 layer, 8 heads, 400 pad size) as the main result.

			AUC (prediction quarter)				
**Ablation**	**Embeddings**	**Features**	*Q1*	*Q2*	*Q3*	*Q4*	Overall
(Phecodes)	All	Phecodes, Neurons	.637	.627	.679	.724	.705
(Labs)	All	Labs, Neurons	.655	.649	.688	.712	.711
(No Neurons)	All	Phecodes, Labs	.646	.628	.682	.732	.708
(No Index Date)	Time, Age, Diagnosis Date	Phecodes, Labs, Neurons	.655	.631	.668	.709	.707
(No Diagnosis Date)	Time, Age, Index Date	Phecodes, Labs, Neurons	.669	.678	.706	.752	.729
(No Diagnosis or Index Date)	Time, Age	Phecodes, Labs, Neurons	.658	.650	.668	.701	.705
(No Age)	Time, Diagnosis Date, Index Date	Phecodes, Labs, Neurons	.664	.659	.692	.714	.719

**Table S.II.3: Sensitivity analysis** with varying start-times, maximum # events, and pad sizes.							
Trajectory Start			*AUC (Prediction Quarter)*				
(Days before Diagnosis)	Max # Events	Pad Size	*Q1*	*Q2*	*Q3*	*Q4*	Overall
0 ± 10	200	200	.644	.646	.683	.709	.705
0 ± 10	800	400	.659	.689	.686	.725	.714
60 ± 10	400	500	.665	.656	.673	.730	.717
90 ± 10	500	500	.656	.651	.663	.693	.719
90± 10	1000	400	.673	.678	.701	.733	.726
90± 30	1000	400	.642	.648	.689	.723	.710
90± 10	800	500	.669	.678	.696	.736	.725
120 ± 10	800	400	.674	.672	.707	.719	.726
120 ± 30	800	400	.667	.675	.710	.723	.726
180 ± 10	800	400	.670	.682	.694	.726	.723
180 ± 20	800	400	.671	.672	.701	.741	.726
180 ± 10	1200	500	.672	.683	.701	.742	.728
540 + 60	1000	400	.667	.674	.699	.723	.724

**Table S.II.4:** Sensitivity Analysis with varying layers, heads, minimum lengths									
Varying Layers and heads					*AUC (by quarter)*				
# Layers	# Heads	Minimum # Events	Max # Events	Pad Size	*Q1*	*Q2*	*Q3*	*Q4*	Overall
1	16	5	500	500	.656	.651	.663	.693	.719
2	8	5	800	300	.675	.677.	.694	.734	.724
Varying Minimum length (all other parameters kept at *default*)									
1	8	20	*800*	*400*	.671	.678	.700	.741	.726
1	8	40	*800*	*400*	.674	.675	.696	.723	.724

Table S.II.5 Sensitivity analyses with varying start times based on patients' time-to-treatment from diagnosis (TTD). Longer TTD corresponds with shorter the look-back windows.

**Start Time**	**Max # Events**	**Pad Size**	*Q1*	*Q2*	*Q3*	*Q4*	Overall
(180– TTD) ± 10 days	800	400	.643	.657	.686	.725	.707
270 days – TTD ± 15 days	800	400	.674	.685	.690	.748	.728
360 days – TTD ± 20 days	800	400	.671	.680	.697	.729	.723

Table S.II.6: Spline Calibration Parameters

**Main Model**				
Parameters\interval	Q1 (0-3)	Q2 (3-6)	Q3 (6-9)	Q4 (9-12)
# Knots	12	9	9	6
Penalty	1.5	2	5.6	2.4
Alternative Model				
# Knots	25	5	4	10
Penalty	5	0.5	0.1	1.3

Figure S.II.4: Confusion matrix/ calibration plots for alternate spline parameters

Q1	VTE+	VTE -
Pred+	380	8478
Pred-	100	7188
**Recall**	**0.792**	

Q2	VTE+	VTE -
Pred+	162	6617
Pred-	58	7538
**Recall**	**0.736**	

Q3	VTE+	VTE -
Pred+	103	4720
Pred-	50	7956
**Recall**	**0.673**	

Q4	VTE+	VTE -
Pred+	82	4692
Pred-	19	6888
**Recall**	**0.812**	

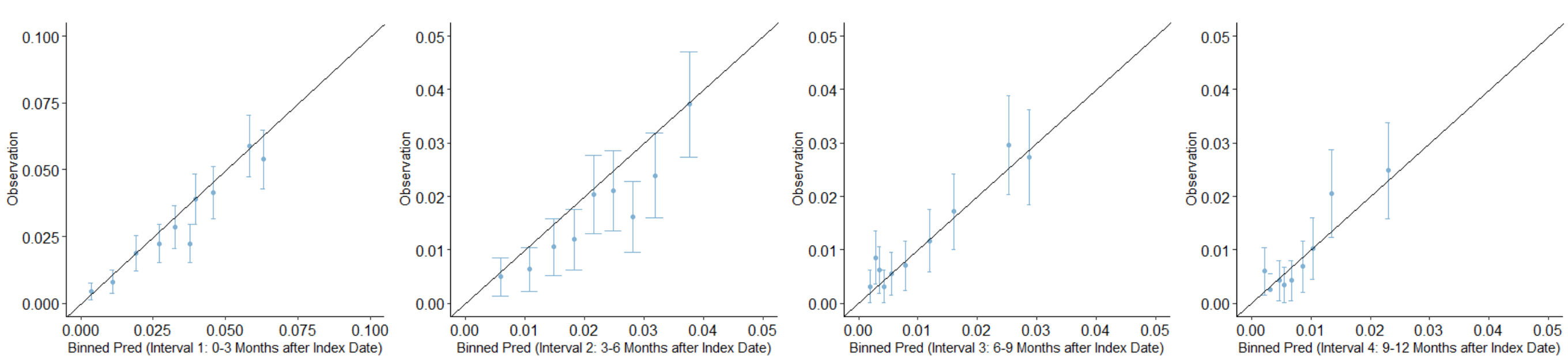

We also recalibrated predictions and generated confusion matrices for the phecodes-only model (from ablation analysis, **Table S.II.2).**

Figure S.II.5: Confusion matrix/calibration plots for Phecode model

Q1	VTE+	VTE -
Pred+	334	7870
Pred-	146	7782
AUC:	0.635	

Q2	VTE+	VTE -
Pred+	183	8401
Pred-	37	5605
AUC:	0.627	

Q3	VTE+	VTE -
Pred+	110	5535
Pred-	43	7141
AUC:	0.679	

Q4	VTE+	VTE -
Pred+	64	3408
Pred-	37	8172
AUC:	0.724	

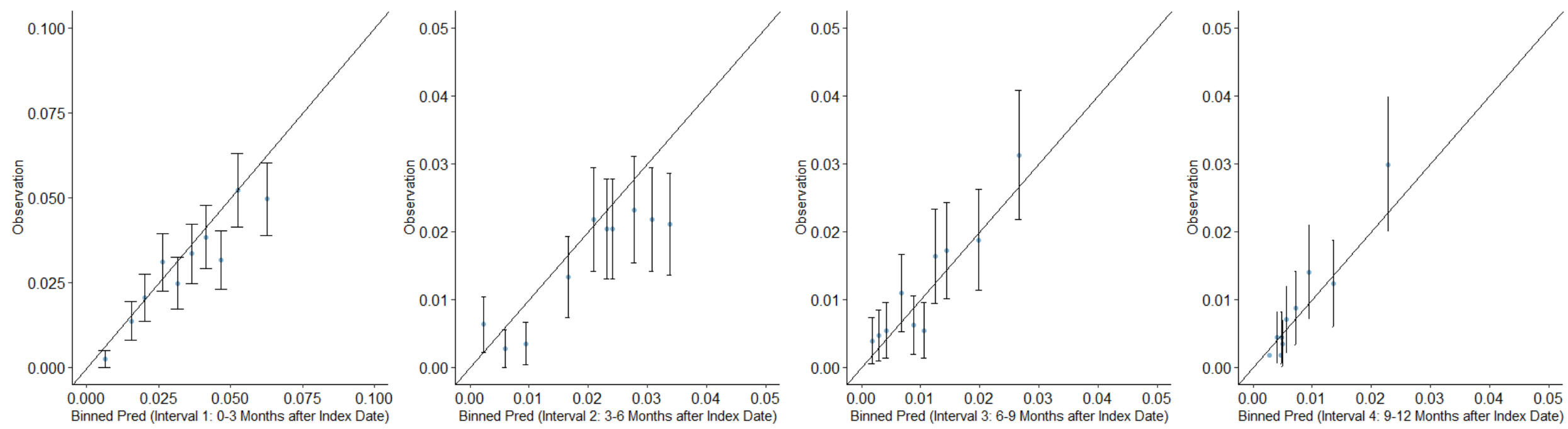

Table S.II.6 Q1 model sensitivity comparison

**Model**	*Cohort*	*AUC* *Q1*	*Q2*	*Q3*	*Q4*
Transformer (Q1 weights)	VA test	.682	.628	.666	.662
Transformer (Q1 weights)	HHS	.683	.604	.647	.656
(Reference from main analysis)					
Transformer	VA test	.682	.679	.707	.765
Transformer	HHS	.683	.703	.709	.738
EHR -CAT	VA test	.634	.645	.613	.617

Supplement III: Subgroup analyses

Supplement III.A: Subgroup analysis in primary test set

Table S.III.1 Model performance stratified by cancer type, cancer stage, and treatment: Stratifications across cancer type, early-intermediate cancer stages, and endocrine/immuno/target therapy were grouped due to small sample size. 95% confidence intervals were provided for the AUC using bootstrap methods.

**Sub-population**			**Test Set AUC [95% CI]**				
Strata			*Q1*	*Q2*	*Q3*	*Q4*	Overall
**Grouped Cancer Type**	*Respiratory System*	*n*	3770	3106	2626	2216	
		Cases (%)	152 (4.0%)	58 (1.9%)	40 (1.7%)	31 (1.5%)	281 (7.5%)
		AUC [CI]	.60 [.55, .64]	.60 [.52, .67]	.67 [.58, .75]	.70 [.61, .78]	.67 [.64, .70]
	Digestive System	*n*	3594	3079	2650	2348	
		Cases (%)	136 (3.8%)	84 (2.7%)	44 (1.7%)	29 (1.2%)	293 (8.2%)
		AUC [CI]	.63 [.59, .67]	.66 [.60, .71]	.73 [.67, .79]	.76 [.67, .85]	.70 [.68, .73]
**Cancer Stage**	*I-III*	*n*	9340	8650	8070	7513	
		Cases (%)	215 (2.3%)	107 (1.2%)	83 (1.0%)	50 (0.7%)	499 (5.3%)
		AUC [CI]	.69 [.66, .72]	.70 [.65, .74]	.71 [.65, .76]	.74 [.66, .81]	.74 [.71, .76]
	Advanced (IV)	*n*	6792	5576	4759	4168	
		Cases (%)	265 (3.9%)	113 (2.0%)	70 (1.5%)	51 (1.2%)	455 (6.7%)
		AUC [CI]	.65 [.61, .68]	.65 [.60, .70]	.70 [.64, .76]	.77 [.72, .82]	.71 [.69, .73]
**Treatment Type**	*Chemotherapy*	*n*	11477	9924	8794	7880	
		Cases (%)	427 (3.7%)	194 (2.0%)	117 (1.3%)	83 (1.1%)	821 (7.2%)
		AUC [CI]	.63 [.60, .66]	.64 [.60, .68]	.70 [.65, .75]	.77 [.73, .82]	.71 [.70, .73]
	Endocrine/ Immuno/Target therapy	*n*	4655	4302	4035	3801	
		Cases (%)	53 (1.1%)	26 (0.6%)	36 (0.9%)	18 (0.5%)	133 (2.8%)
		AUC [CI]	.77 [.72, .82]	.71 [.61, .82]	.71 [.63, .79]	.66 [.53, .80]	.72 [.68, .76]

Table S.III.2: Demographic subgroup analysis stratified by age, race/ethnicity, and sex subpopulations. 95% confidence intervals were provided for the AUC using bootstrap methods.

**Sub-population**			**Test Set AUC [95% CI]**				
Strata			*Q1*	*Q2*	*Q3*	*Q4*	Overall
**Age**	*75 and over*	*n*	2893	2480	2208	1967	
		Cases (%)	67 (2.3%)	31 (1.3%)	29 (1.3%)	16 (.8%)	143 (4.9%)
		AUC [CI]	.69 [.64, .74]	.66 [.57, .76]	.68 [.58, .79]	.70 [.56, .84]	.71 [.67, .75]
	65 -74	*n*	7016	6172	5507	5034	
		Cases (%)	215 (3.1%)	103 (1.7%)	62 (1.1%)	38 (.8%)	418 (6.0%)
		AUC [CI]	.68 [.65, .71]	.66 [.61, .71]	.71 [.65, .77]	.80 [.74, .85]	.74 [.72, .76]
	Under 65	*n*	6226	5575	5114	4680	
		Cases (%)	198 (3.2%)	86 (1.5%)	62 (1.2%)	47 (1.0%)	393 (6.3%)
		AUC [CI]	.67 [.63, .71]	.71 [.66, .76]	.72 [.66, .78]	.76 [.69, .83]	.74 [.71, .76]
**Race**	*Black*	*n*	3295	2944	2704	2477	
		Cases (%)	96 (3.0%)	39 (1.3%)	44 (1.6%)	29 (1.2%)	208 (6.3%)
		AUC [CI]	.72 [.67, .77]	.69 [.61, .78]	.68 [.61, .75]	.83 [.76, .91]	.73 [.70, .76]
	Non-White	*n*	4411	3939	3603	3300	
		Cases (%)	121 (2.7%)	65 (1.7%)	53 (1.5%)	35 (1.1%)	274 (6.2%)
		AUC [CI]	.71 [.67, .75]	.68 [.62, .74]	.67 [.60, .73]	.83 [.76, .90]	.73 [.70, .75]
	White	*n*	11721	10287	9226	8381	
		Cases (%)	359 (3.1%)	155 (1.1%)	100 (1.1%)	66 (.8%)	680 (5.8%)
		AUC [CI]	.67 [.64, .69]	.68 [.64, .72]	.73 [.68, .78]	.73 [.67, .79]	.74 [.72, .75]
**Sex**	*Female*	*n*	790	744	711	679	
		Cases (%)	16 (2.0%)	6 (.8%)	6 (.8%)	7 (1.0%)	35 (4.4%)
		AUC [CI]	.77 [.66, .89]	.76 [.63, .88]	.82 [.65, 1]	.76 [.61, .91]	.75 [.68, .82]
	Male	*n*	15432	13482	12118	11002	
		Cases (%)	464 (3.0%)	214 (1.6%)	147(1.2%)	94 (.9%)	919 (6.0%)
		AUC [CI]	.67 [.65, .70]	.68 [.65, .71]	.70 [.66, .74]	.77 [.72, .81]	.73 [.72, .75]

Supplement III.B: Subgroup analysis in HHS external validation cohort

Table S.III.3: Demographic subgroup analysis for HHS external validation cohort stratified by age, race/ethnicity, and sex subpopulations matching that of the primary VA test set. 95% confidence intervals were provided for the AUC using bootstrap methods.
Age was stratified slightly differently than that of the primary test set: because there were far fewer patients over age 65, the *65-74* and *75 and over* subgroups were combined. The *under 55* subgroup was created only in HHS to highlight model performance on younger patients, who were underrepresented in the primary test set.

**Sub-population**			**External Validation Set AUC [95% CI]**				
Strata			*Q1*	*Q2*	*Q3*	*Q4*	Overall
**Age**	*65 and over*	*n*	1342	1113	918	748	
		Cases (%)	61 (4.6%)	29 (2.6%)	8 (0.9%)	4 (0.5%)	102 (7.6%)
		AUC [CI]	.73 [.66, .78]	.69 [.59, .78]	.67 [.34, .96]	.88 [.75, .96]	.78 [.74, .82]
	Under 65 (inclusive of <55)	*n*	8410	7424	6326	5214	
		Cases (%)	317 (3.8%)	155 (2.1%)	85 (1.3%)	62 (1.2%)	619 (7.4%)
		AUC [CI]	.68 [.65, .70]	.71 [.67, .74]	.71 [.66, .76]	.73 [.65, .79]	.73 [.71, .75]
	Under 55	*n*	4822	4300	3683	3055	
		Cases (%)	192 (4.0%)	90 (2.1%)	44 (1.2%)	27 (0.9%)	353 (7.3%)
		AUC [CI]	.67 [.63, .70]	.71 [.66, .76]	.72 [.65, .79]	.78 [.70, .85]	.75 [.72, .77]
**Race**	*Black*	*n*	2569	2200	1836	1467	
		Cases (%)	130 (5.1%)	59 (2.7%)	32 (1.7%)	28 (1.9%)	249 (9.7%)
		AUC [CI]	.67 [.63, .72]	.68 [.62, .73]	.69 [.59, .77]	.70 [.60, .79]	.72 [.69, .75]
	Non-White	*n*	8277	7294	6259	5177	
		Cases (%)	310 (3.8%)	143 (2.0%)	82 (1.3%)	50 (1.0%)	585 (7.1%)
		AUC [CI]	.69 [.66, .72]	.70 [.65,.73]	.70 [.65,.76]	.74 [.66, .80]	.74 [.72, .76]
	White	*n*	1475	1243	985	785	
		Cases (%)	68 (4.6%)	41 (3.3%)	11 (1.1%)	16 (2.0%)	136 (9.2%)
		AUC [CI]	.64 [.66, .73]	.72 [.65, .79]	.78 [.62, .89]	.73 [.52, .86]	.73 [.69, .77]
**Sex**	*Female*	*n*	5547	4953	4301	3623	
		Cases (%)	206 (3.7%)	93 (1.9%)	55 (1.3%)	34 (0.9%)	388 (7.0%)
		AUC [CI]	.69 [.66, .73]	.73 [.68, .78]	.73 [.66, .80]	.77 [.67, .84]	.76 [.73, .78]
	Male	*n*	4205	3584	2943	2339	
		Cases (%)	172 (4.1%)	91 (2.5%)	38 (1.3%)	32 (1.4%)	333 (7.9%)
		AUC [CI]	.67 [.63, .70]	.66 [.61, .71]	.67 [.59, .74]	.70 [.58, .79]	.72 [.69, .74]

**Table S.III.4.** Breast cancer subgroup performance in HHS validation cohort					
	**AUC [95% CI]**				
	*Q1 (*incidence)	*Q2*	*Q3*	*Q4*	Overall (prevalence)
n	2180	2034	1873	1663	
Cases (%)	39 (1.8%)	18 (.9%)	22 (1.2%)	10 (.6%)	89 (4.1%)
AUC [CI]	.66 [.57, .74]	.77 [.65, .86]	.71 [.60, .80]	.86 [.74, .95]	.73 [.68, .78]

Supplement IV: Temporal diagnostics

Supplement IV.A: Cross-sectional diagnostics

Test set predicted probabilities are compared with time-to-VTE at each interval to check potential temporal bias. Binary predictions post-calibration and thresholding are also compared with time-to VTE.

Figure S.IV.1: *(Top)* Predicted (raw) probabilities of VTE patients (all sets) compared to day of occurrence post-treatment index date (stratified by in lookforward window). (*Bottom*) Histograms of days-to-VTE for each 3-month prediction window, separated by positive and negative predictions (post-calibration and classification into pred+/pred-).

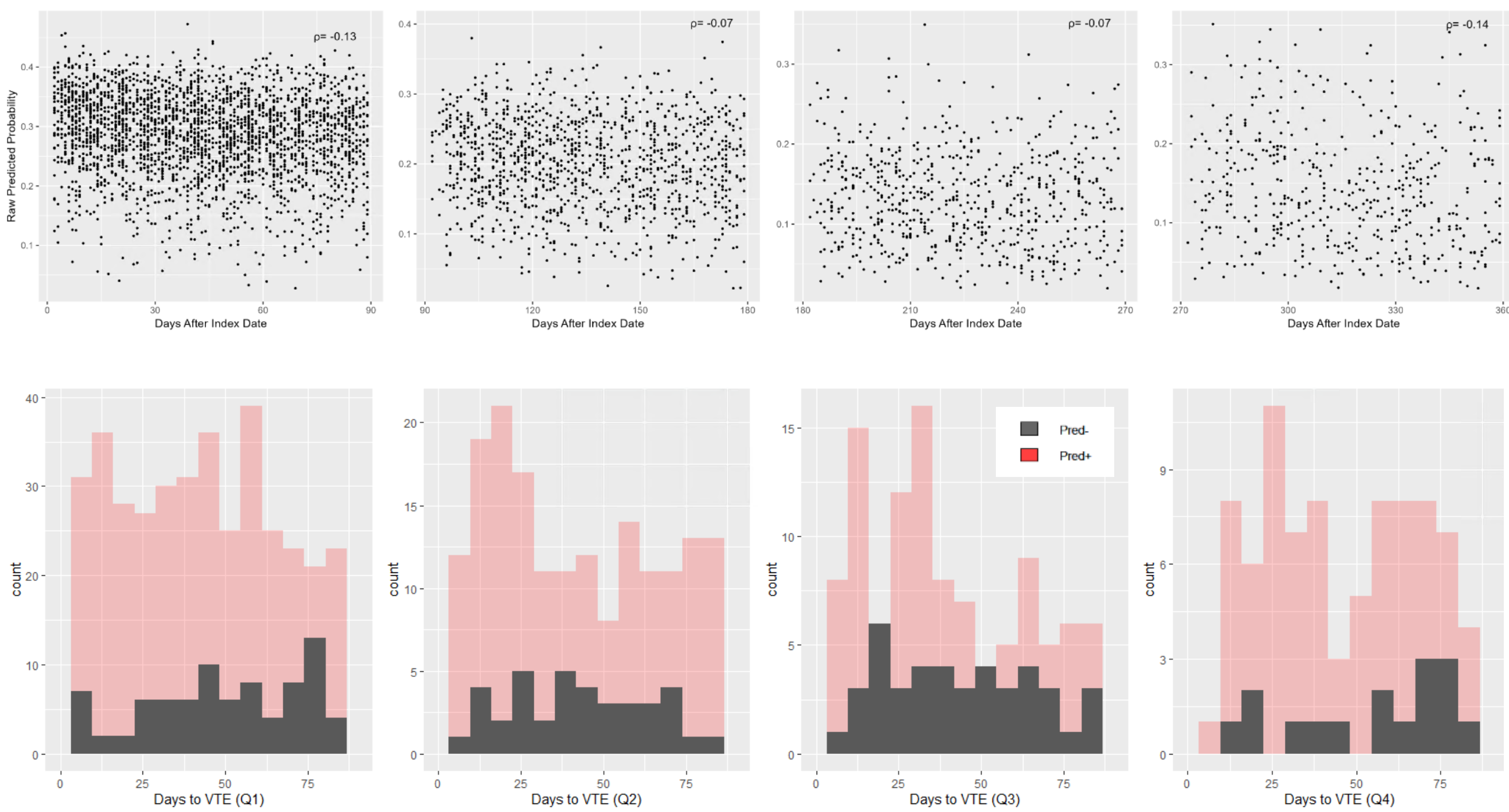

Filtering times-to-treatment in the test set does not change the performance by much, indicating that the performance is not driven by abnormally lengthy wait-times that could be indicative of out-of-VA-network treatment switching.

Table S.IV.1: Cross- Sectional temporal validation for test set (2011-2020): characteristics and performance results of filtering time-to-treatment-from-diagnosis (TTD) by 120 days.

	**Filtered (Time-to-treatment < 4 months)**			
	Quarter	*n (% of total)*	*Cases (% of n)*	*AUC*
**2011-2020**	*Q1*	13581 (84%)	444 (3.3%)	0.659
	Q2	11853 (83%)	198 (1.7%)	0.661
	Q3	10615 (83%)	137 (1.3%)	0.702
	Q4	9631 (85%)	91 (0.9%)	0.773
	Overall		803	0.729

We conduct additional sensitivity analysis by examining stratified ranges of index dates for systemic treatment initiation.

When VTE incidence rates for both primary (index dates 2011-2020) and temporal validation (2021-2022) cohorts are considered, the rates are mostly stable from 2011-2019 but a noticeable anomaly is present in 2020-2021 (**Figure S.IV.2**). This may be partly attributable to the COVID-19 pandemic, which was associated with a reduction in the number of patients treated (**Figure S.IV.3**) as well as times to treatment (**Figure S.IV.4),** data leakage, or a combination of both.

Figure S.IV.2: VTE incidence rate (all quarters combined) for both the primary cohort and the temporal validation set (split indicated by the black dashed line). The grey dotted line indicates the onset of the COVID-19 pandemic starting in 2020.

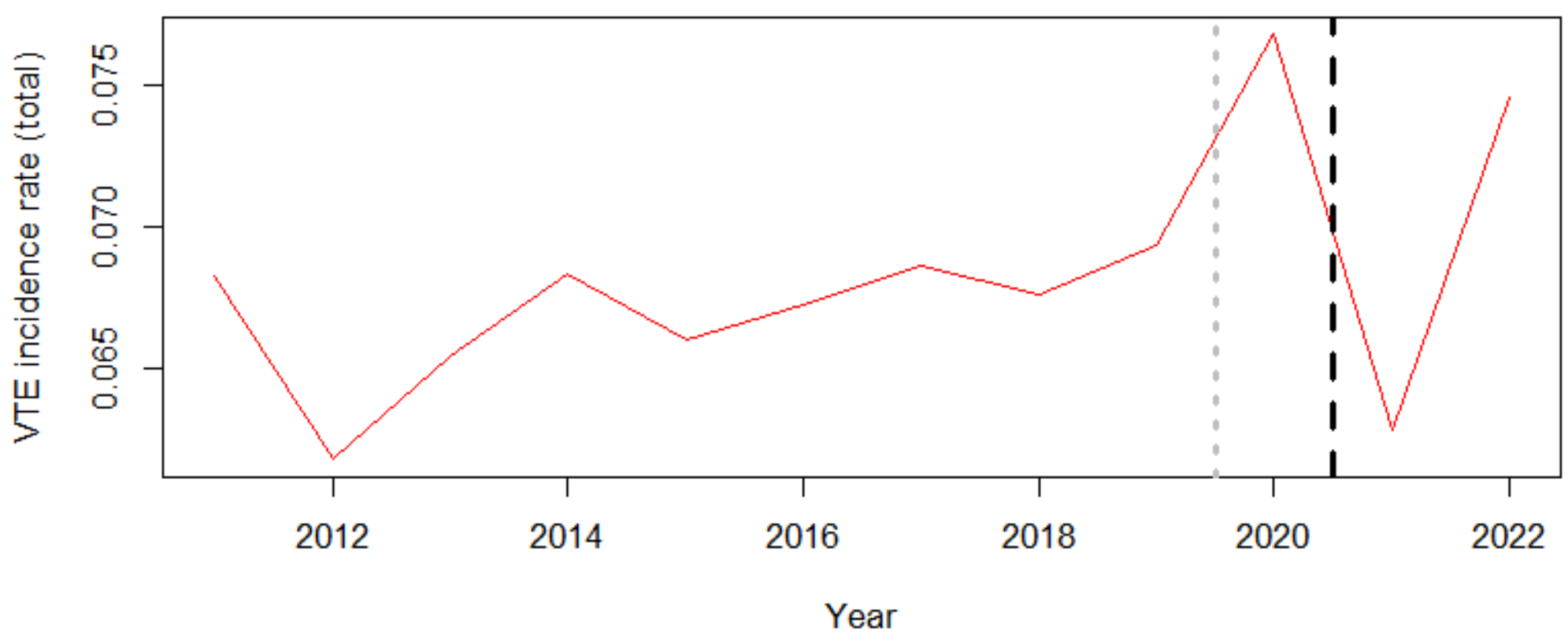

Figure S.IV.3: Frequency of index dates (start times of systemic cancer treatment) for both the primary cohort and the temporal validation set (split indicated by the dashed line)

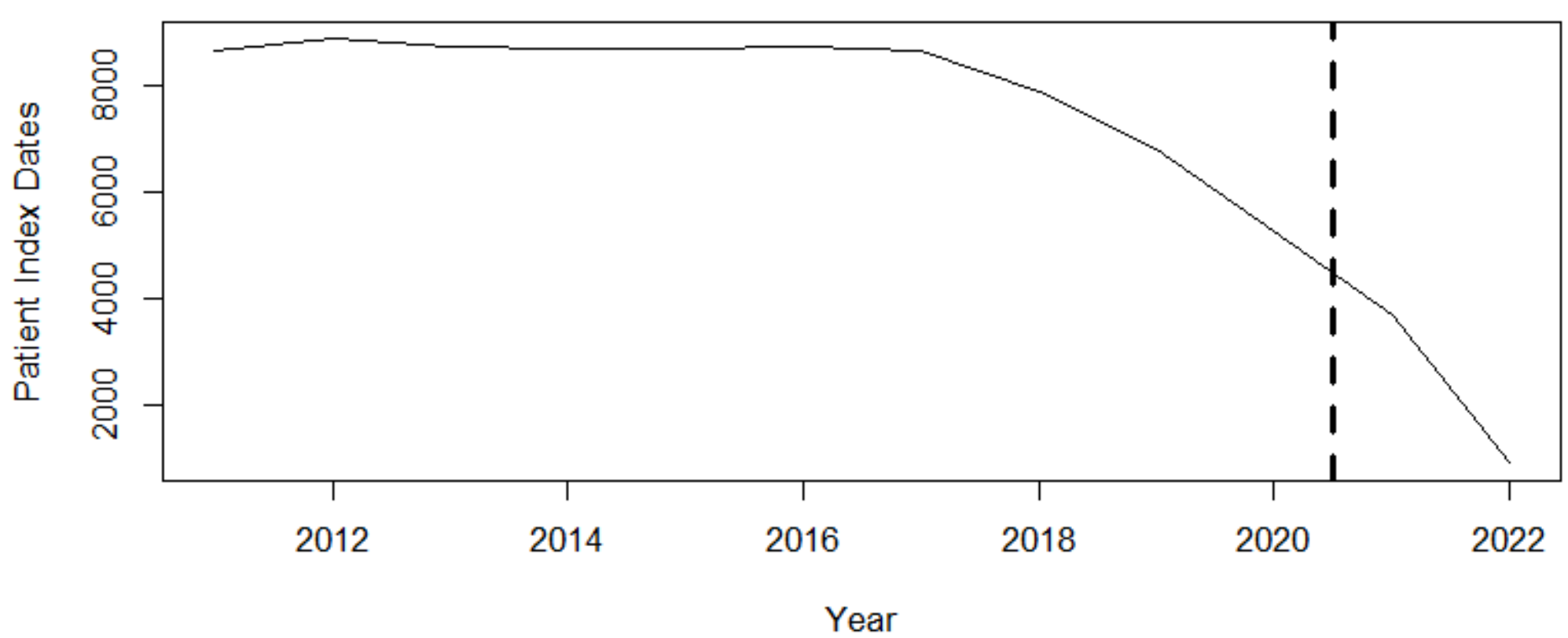

The COVID-19 Pandemic, which was a public health emergency of international concern from March 2020 - May 2023 has systematically delayed cancer prognosis and treatment. Such delays may be linked to changing VTE etiology over time. We examine performance in the first year of COVID-19 (2020). The proportion of patients with index dates in 2020 are lower in proportion (6-7%) compared to the proportional 10% possibly because treatments are delayed until 2021, which will further contribute to the bias in the validation set (2021-2022). Q1-Q2 performance is higher but Q3-Q4 performance in 2020 is considerably lower than the overall (**Table S.IV.2**).

Table S.IV.2: Cross- Sectional temporal validation for test set (2020)

Quarter	*n (% of total)*	*Cases (% of n)*	*AUC*
Q1	1041 (6%)	34 (3.3%)	0.700
Q2	913 (6%)	12 (1.3%)	0.712
Q3	846 (7%)	15 (1.8%)	0.680
Q4	773 (7%)	11 (1.4%)	0.708
Overall		72	0.718

Performance prior to the pandemic (index dates 2011-2019) of the study window appears to be consistent with the overall results.

Table S.IV.3: Cross- Sectional temporal validation for test set (2011-2019)

Quarter	*n (% of total)*	*Cases (% of n)*	*AUC*
Q1	15091 (94%)	446 (3.0%)	0.677
Q2	13313 (93%)	208 (1.6%)	0.679
Q3	11983 (93%)	138 (1.2%)	0.710
Q4	10908 (93%)	90 (0.8%)	0.771
Overall		882	0.735

Supplement IV.B: Temporal validation diagnostics

Because records become sparser as index dates approaches the end of the study window (June 2023), we exclude patients after the median index date in 2022 (April 25). Many patients who develop VTE do not yet have records of these eventual outcomes.

The temporal validation set from 2021-2022 is contained entirely during the extenuating circumstances of the COVID-19 pandemic and as such is subject to bias from delayed and protracted times to treatment. The mean time to treatment from diagnosis (TTD) is 71 (IQR: [27, 98]) days in the Test set and 78 [28,103] days in the TV set (**Figure S.IV.4**). The mean difference is 7.7 ± 2.7 days (two sample t-test). When considering only patients who develop VTE, TTD is consistent and lower than total in the Test set (43 [22,70] days), but more aberrant and heterogeneous in the TV cohort in the later quarters, reaching up to a median of 67 [32, 129] days in Q4.

Figure S.IV.4: Mean times to treatment from diagnosis (TTD) for the test and validation sets at given prediction quarter., for all patients and patients who develop VTE at the given quarter. Effects of the 120-day TTD filter are also shown.

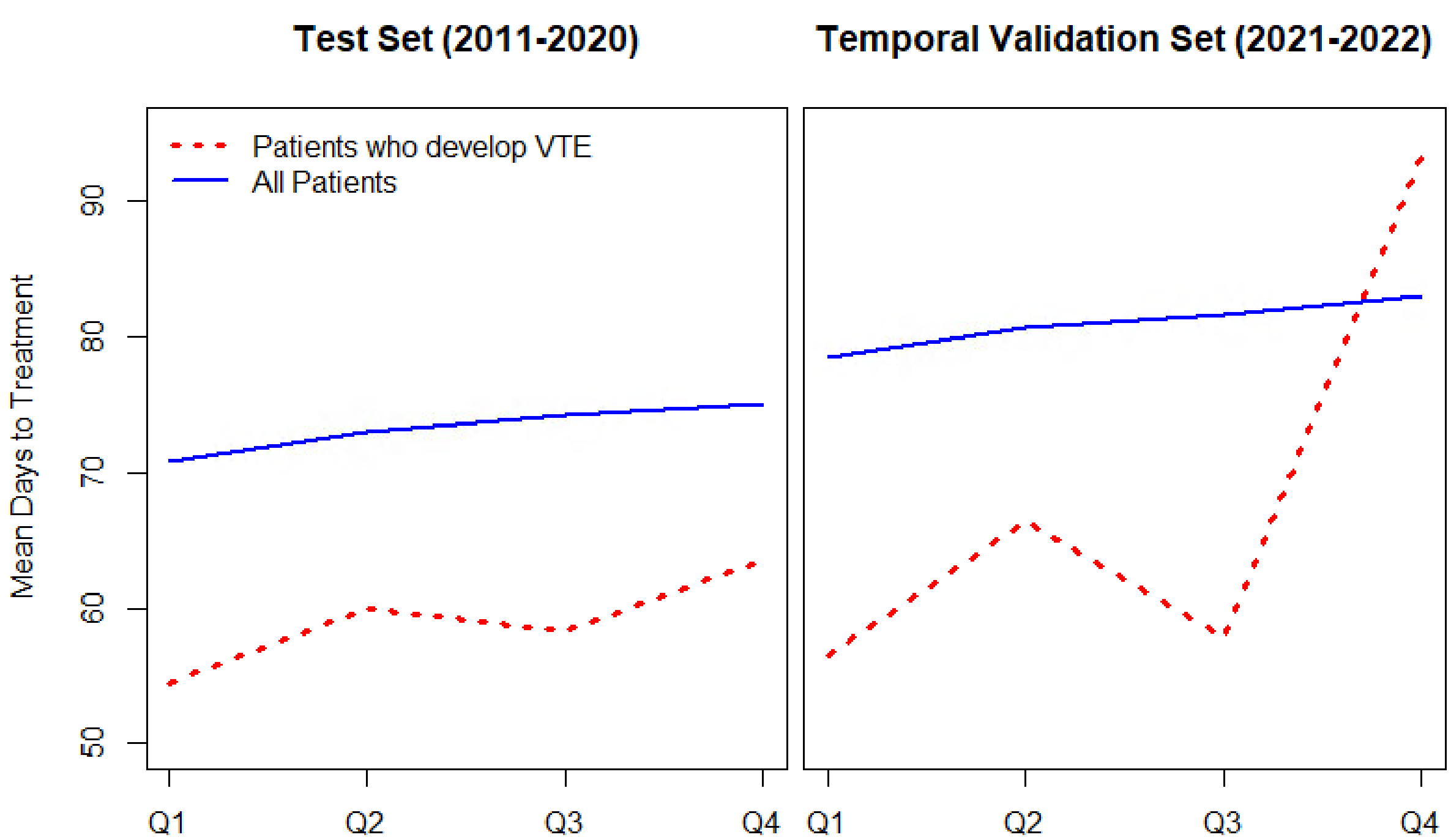

The resultant bias from lengthier times to treatment in TV set may be controlled by restricting patients with TTD under 120 days (**Figure S.IV.4**), This filter reduces the total sample size by 19% (4098 to 3303) in the TV set. Kaplan Meier curves are shown in **Figure S.VI.2.** The difference in Q3 may be attributed to the abnormally low incidence rate in the TV set.

While excluding patients with longer times to treatment is not advisable for general VTE prediction models, this filter is applied here to demonstrate that the proposed model maintains

consistent performance despite COVID-19-related disruptions. This underscores the importance of timely systemic treatment. Performance of the transformer model on the unfiltered TV set is shown in **Table S.IV.4.**

Table S.IV.4: Validation set performance on total temporal validation cohort, without filtering by time-to-treatment, from Jan. 2021-Apr. 2022

Quarter	*n*	*Cases (%)*	*AUC*
Q1	4098	146 (3.6%)	0.663
Q2	3616	57 (1.6%)	0.655
Q3	3327	17 (0.5%)	0.795
Q4	3104	23 (0.7%)	0.681
Overall		243	0.759

Supplement V: Details of input features

**Table S.V.1 Trajectory characteristics of full phecode and lab features** for the full dataset in the primary cohort (5 years before and 2 years after index date).		
	Non-VTE	VTE
**Phecode Characteristics**		
# Events, median (IQR)	292 [190, 451]	374 [255, 558]
Time length (years), median (IQR)	6.3 [5.3, 6.8]	5.0 [4.6, 5.3]
**Lab Characteristics**		
# Events, median (IQR)	562 [359, 863]	793 [550, 1160]
Time length (years), median (IQR)	5.8 [4.9, 6.5]	5.4 [4.8, 6.3]

Figure S.V.1 Relative frequencies of phecode categories for the full dataset in the primary cohort , sorted from most frequent to least.

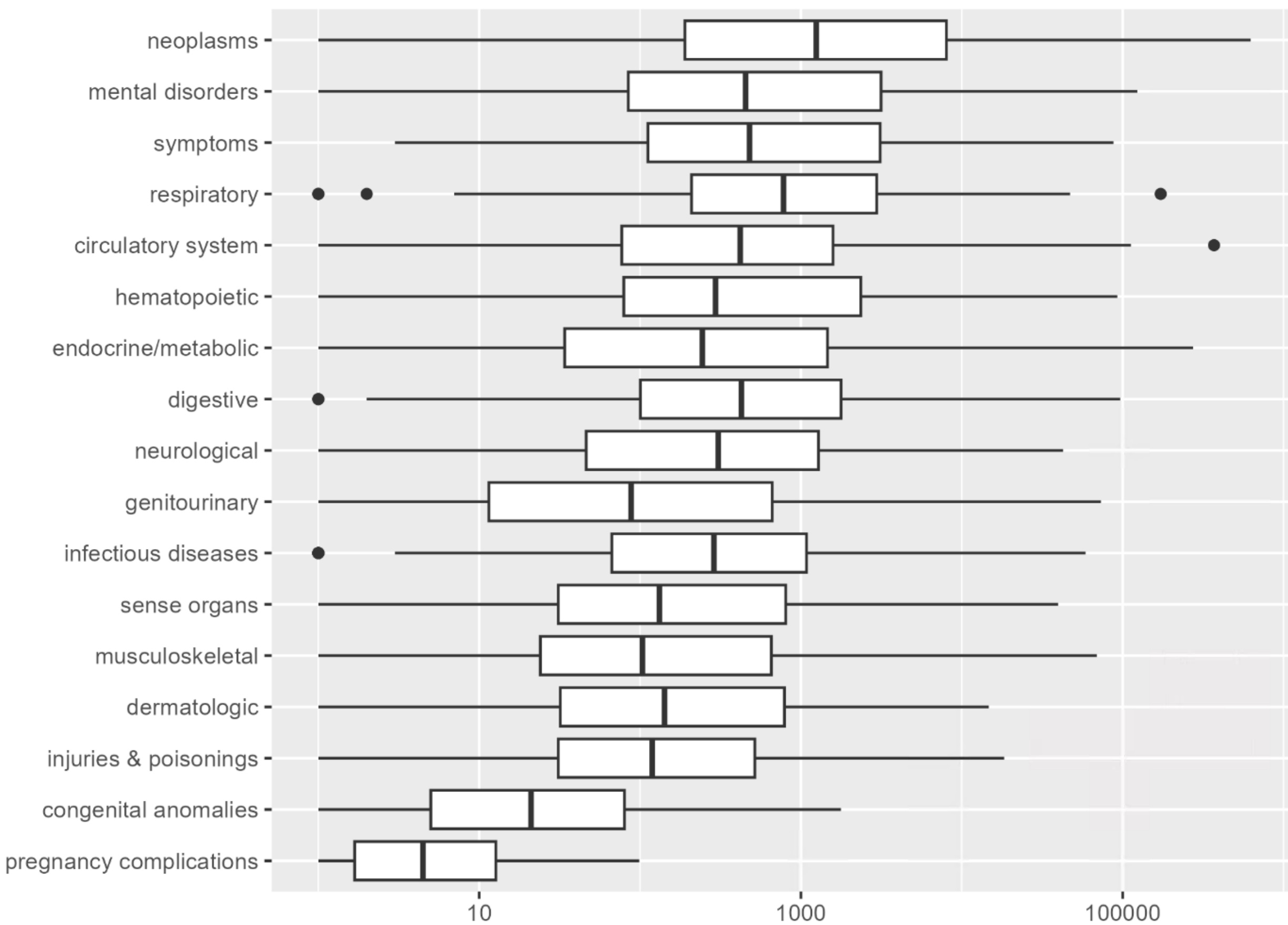

**Table S.V.2:** Laboratory test Characteristics of primary cohort					
	Lab	*# subjects*	*# per subject, total Mean [IQR]*	*# per subject in ref. range, Mean [IQR]*	*LOINC Code(s)*
CBC	Hemoglobin (mass/volume in blood)	79455	7.1 [4,8]	3.1 [1,4]	718-7
	Platelet (#/volume in blood by auto. Count)	79455	6.2 [4,8]	4.6 [3,6]	777-3
	RDW (red cell distribution width)	76631	12.6 [6,17]	6.9 [3,10]	788-0, 30385- 9
	WBC (leukocytes) (#/volume in blood by auto. Count)	79455	12.7 [7,17]	8.4 [4,11]	6690-2
CMP	Albumin	80259	7.0 [4,8]	4.8 [3,6]	2862-1, 61151-7, 1751-7
	Alkaline Phosphatase	80678	3.7 [4,4]	3.2 [2,4]	1783-0, 6768-6
	ALT	80792	9.4 [4,12]	7.6 [4,11]	1742-6, 1743-4, 1744-2, 76625-3
	AST	79462	9.3 [4,12]	7.4 [4,10]	1920-8, 30239-8
	Bilirubin	80677	4.3 [4,4]	3.7 [3,4]	1975-2, 42719-5, 59828-4
	BUN	80722	13.2 [7,18]	9.0 [4,12]	12962-7, 12963-5, 6299-2 *3094-0*
	Calcium	80560	9.3 [4,12]	6.8 [4,9]	17861-6, 49765-1
	Carbon Dioxide	78517	3.4.2 [4,4]	3.4 [2,4]	19223-7, 2026-3, 2027-1, 2028-9, 20565-8, 2069-3,
	Chloride	79707	6.3 [4,8]	4.5 [3,6]	2075-0, 41649-5, 41650-3
	Creatinine	80541	7.5 [4,8]	5.3 [3,7]	21232-4, 2160-0, 38483-4
	Glucose	78481	19.6 [6,20]	6.3 [2,8]	2339-0, 2340-8, 2341-6, 2345-7, 32016-8, 41651-1, 41652-9, 41653-7, 74774-1
	Potassium	80807	5.8 [4,8]	4.8 [3,6]	2823-3, 6298-4, 32713-0, 39789-3, 41656-0, 77142-8
	Protein	77387	6.7 [4,8]	5.0 [3,7]	2885-2, 13537-6, 20577-3
	Sodium	80806	4.3 [4,4]	3.3 [2,4]	2947-0, 2951-2, 32717-1, 39791-9, 41657-8, 77139-4

Supplement VI: Attribution analysis

Figure S.VI.1: Attribution analysis: Attribution weights for the TF model at each prediction interval calculated from layer integrated gradients. Attributions were calculated for merged trajectories of disease and lab tokens. Frequencies with counts of at least 10 from layer integrated gradients were retained for each quarter. Secondary malignancy of the liver indicates metastatic liver involvement, most commonly originating from colorectal, pancreatic, bile duct, or lung cancers. From Q1-Q4, the attribution of brain cancer increases, whereas the influence of secondary liver malignancy, high platelet count, low albumin, and lung cancer decreases. A prior history of DVT (deep vein thrombosis) ranks highly in attribution during the first quarter but is absent from later time intervals.

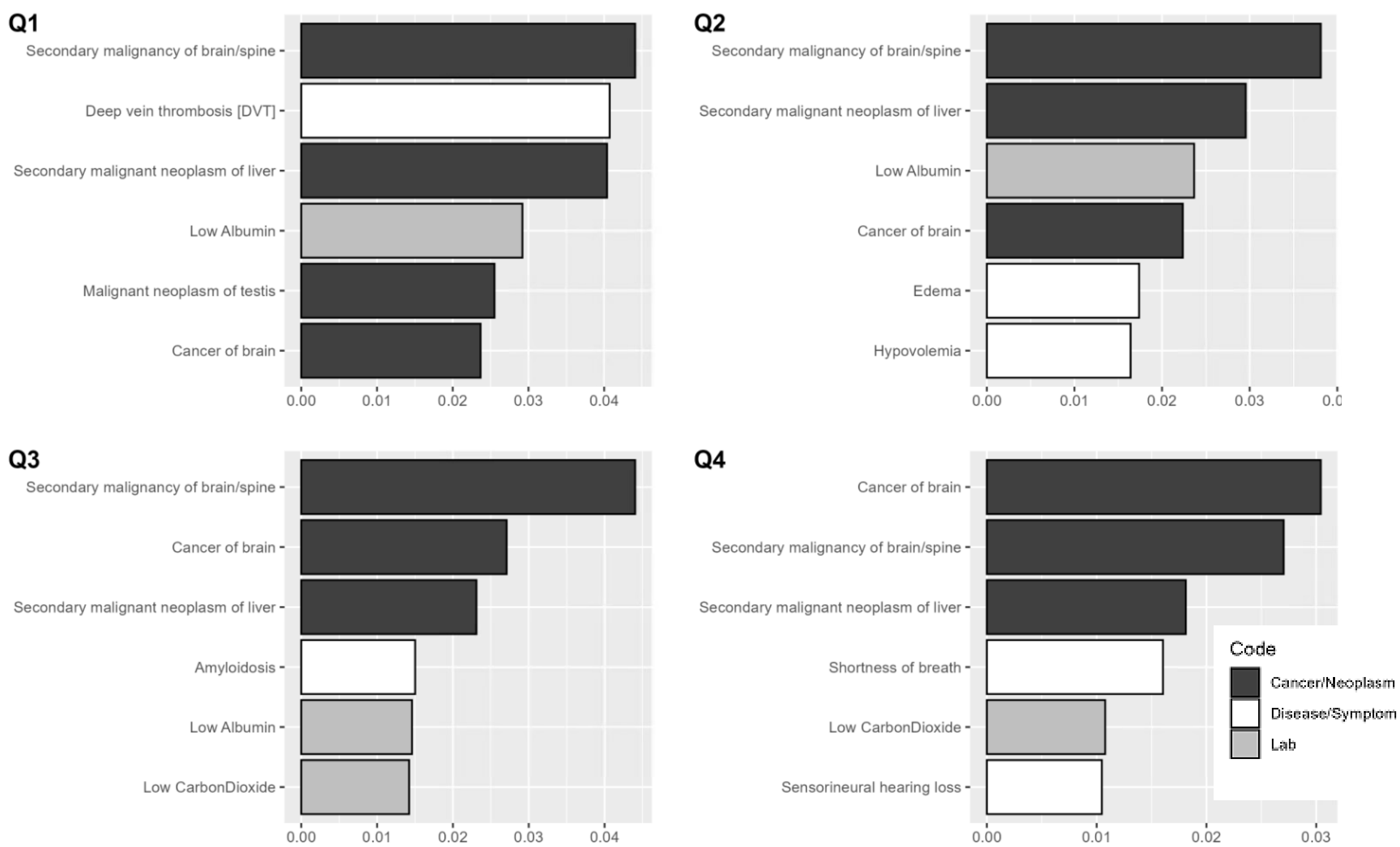

Figure S.VI.2: Trends of top attributions Top attribution weights plotted over time, split into those that are increasing and decreasing across the four intervals.

Decreasing Over Time

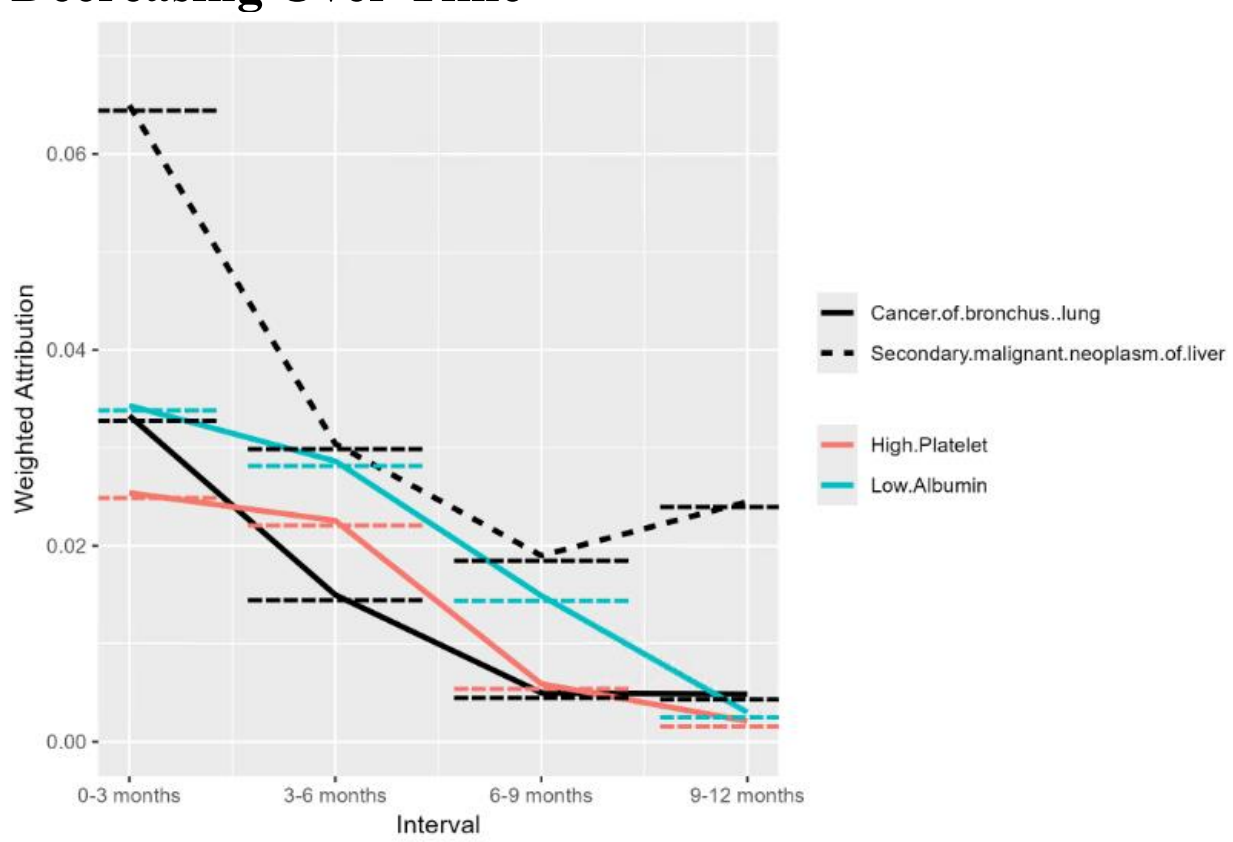

Increasing Over Time

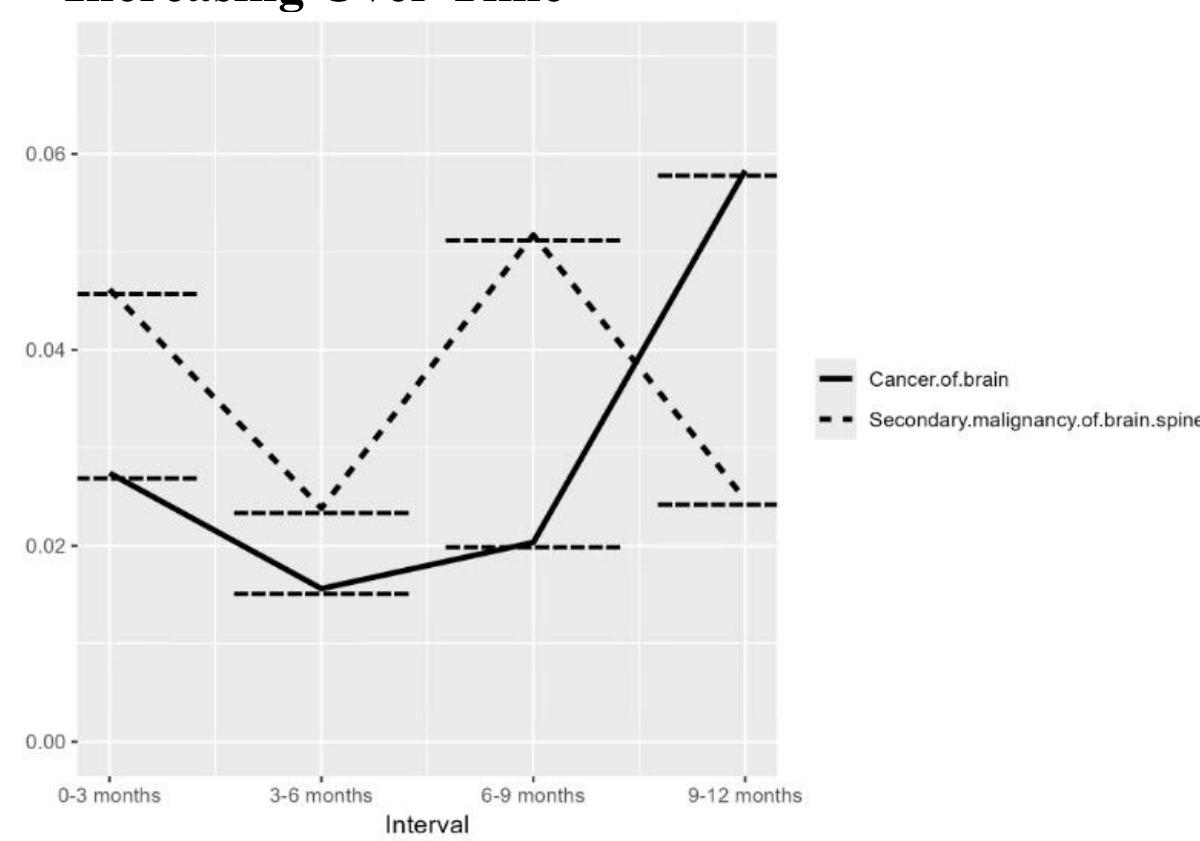

Supplement VII: Competing risks analysis

Figure S.VII.1:. Cause-specific AUC (CS-AUC) for VTE risk for each prediction window in the test set, accounting for the competing risk of death (not attributable to VTE). CS-AUCs were calculated at 30, 45, 60, 75, 90 days into the prediction window.

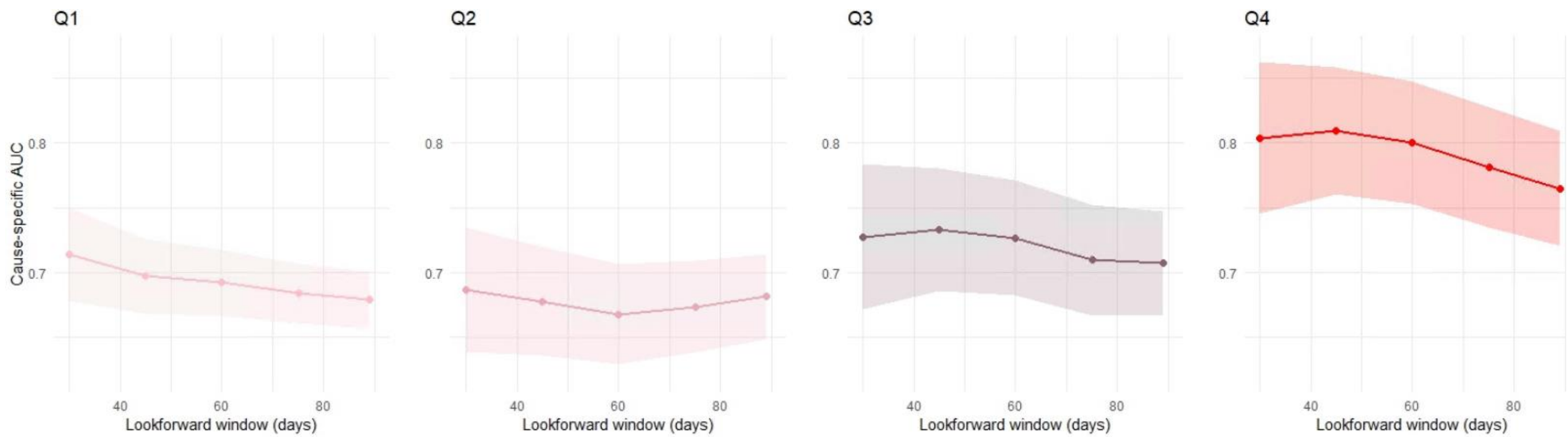

Figure S.VII.2: Calibration plots for recalibrated predictions using CIFs for VTE as observed probability bins. CIFs are estimated using Fine-Gray competing risks with a smooth spline of the logit prediction, as in the recalibration model, across different time-bins of 15 day intervals.

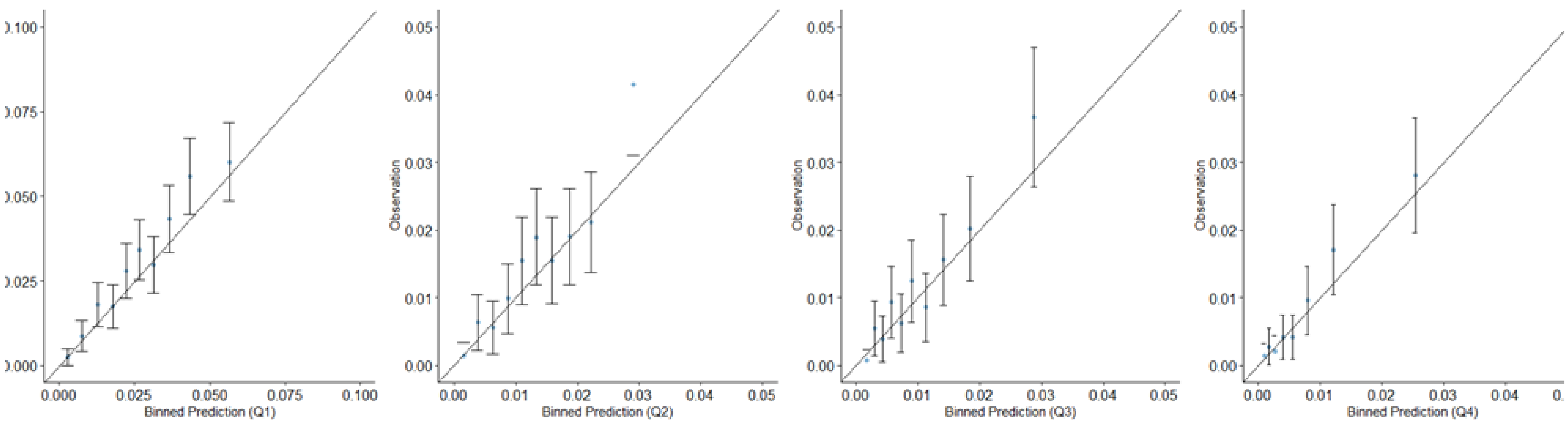

Supplement VIII: Kaplan Meier curves

Figure S.VIII: Kaplan-Meier curves for TF and EHR-CAT model. Cumulative incidence plots, split by VTE and non-VTE, across time for the four prediction intervals for the TF and EHR-CAT models. VTE incidence dates, dates of dropout, or the maximum time-window censor (90 days) are used as event dates

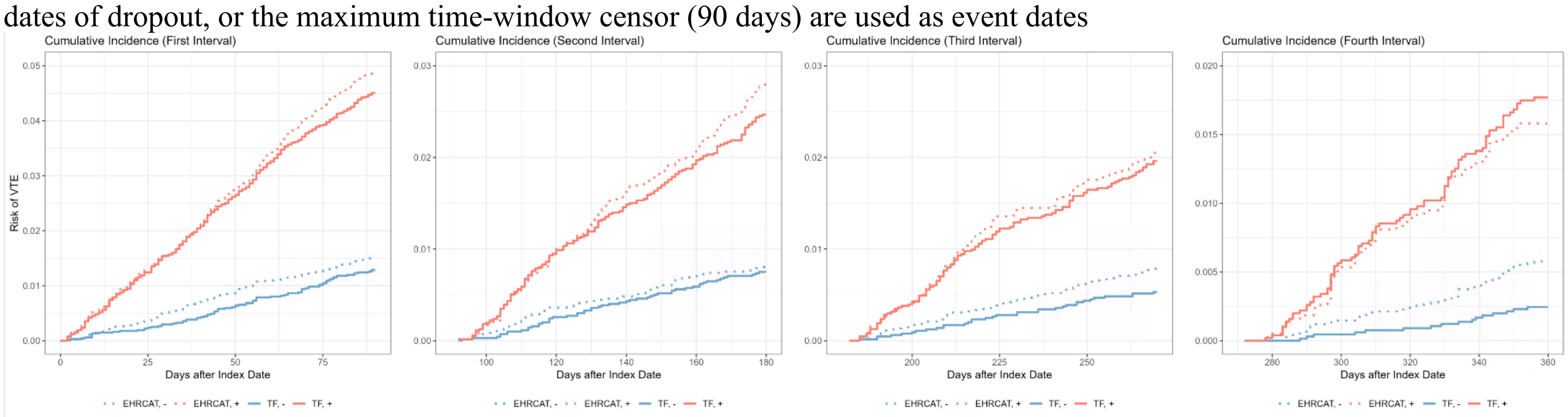

Supplement IX: Efficiency metrics and operational workload quantification

Table S.IX.1 Precision, recall, NNE (number needed to evaluate among positive predictions) and NNS-V (number needed to screen to prevent one positive case of VTE) **(**assuming absolute risk reduction (ARR) of 5% for on-treatment DOAC use for all quarters (Li et al, 2019)) for transformer, EHR-CAT, and Khorana Score (KS) models.

**Quarter**	**Metric**	**Model**		
		Transformer	EHR-CAT	KS
Q1	*Precision*	.042	.046	.066
	Recall	.82	.74	.19
	(NNE)	*24*	*22*	*15*
	(NNS-V)	*819*	*904*	*3469*
Q2	*Precision*	.023	.026	.034
	Recall	.76	.70	.15
	(NNE)	43	38	29
	(NNS-V)	*1693*	*1848*	*8892*
Q3	*Precision*	.019	.019	.020
	Recall	.78	.59	.09
	(NNE)	53	53	50
	(NNS-V)	*2156*	*2851*	*18327*
Q4	*Precision*	.017	.014	.017
	Recall	.84	.56	.14
	(NNE)	*59*	*71*	*59*
	(NNS-V)	*2748*	*4099*	*16687*

Where NNS-V = 1/ (3-month prevalence * recall * ARR), assuming all cases have not been priorly diagnosed. NNE = 1/precision.

Figure S.IX.1 – Clinical screening workflow diagram within a prediction-augmented infrastructure. Modeled after PASI, the system ingests patient data nightly through extract, transform, and load (ETL) processes, applies NLP algorithms to extract clinical variables from unstructured notes, generates risk scores using the transformer model, and displays ranked patient lists through a user interface. Clinicians review flagged patients, confirm eligibility, and initiate appropriate follow-up with standardized documentation templates, enabling consistent workflows and measurable labor-time estimates across sites. Complex or borderline cases are escalated to additional review through a queue-based system with structured summary reports.

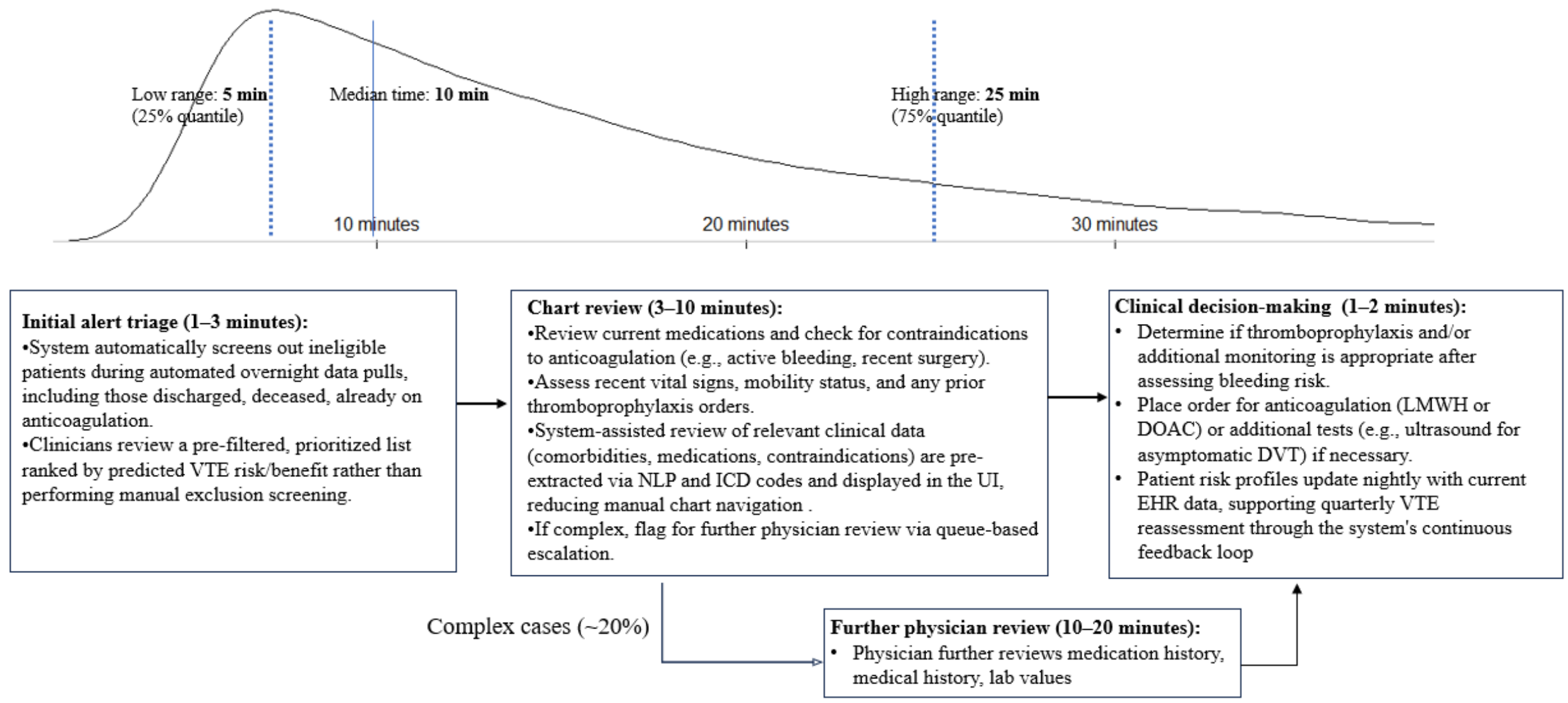

Table S.IX.2 Operational workload analysis for the transformer model within prediction-augmented clinical workflow assuming 10 (IQR: 5-25) minutes per true positive case of VTE.

	Labor-time per VTE detected (LTV) [IQR]
Q1	4.0 [2.0, 9.9]
Q2	7.3 [3.6, 18.1]
Q3	8.8 [4.4, 21.9]
Q4	9.8 [4.9, 24.5]
